# Molecular Detection and Distribution of Six Medically Important *Vibrio* spp. in Selected Freshwater and Brackish Water Resources in Eastern Cape Province, South Africa

**DOI:** 10.3389/fmicb.2021.617703

**Published:** 2021-06-02

**Authors:** Oluwatayo E. Abioye, Ayodeji Charles Osunla, Anthony I. Okoh

**Affiliations:** ^1^SAMRC Microbial Water Quality Monitoring Centre, University of Fort Hare, Alice, South Africa; ^2^Applied and Environmental Microbiology Research Group, Department of Biochemistry and Microbiology, University of Fort Hare, Alice, South Africa; ^3^Department of Microbiology, Obafemi Awolowo University, Ife, Nigeria; ^4^Department of Microbiology, Adekunle Ajasin University, Akungba-Akoko, Nigeria; ^5^Department of Environmental Health Sciences, University of Sharjah, Sharjah, United Arab Emirates

**Keywords:** pathogenic *Vibrio* spp., temperature, salinity, public-health, brackish-water, freshwater

## Abstract

Water resources contaminated with pathogenic *Vibrio* species are usually a source of devastating infection outbreaks that have been a public health concern in both developed and developing countries over the decades. The present study assessed the prevalence of six medically significant *Vibrio* species in some water resources in Eastern Cape Province, South Africa for 12 months. We detected vibrios in all the 194 water samples analyzed using polymerase chain reaction (PCR). The prevalence of *Vibrio cholerae*, *Vibrio mimicus*, *Vibrio fluvialis*, *Vibrio vulnificus*, *Vibrio alginolyticus*, and *Vibrio parahaemolyticus* in freshwater samples was 34, 19, 9, 2, 3, and 2%, and that in brackish water samples was 44, 28, 10, 7, 46, and 51%, respectively. The population of the presumptive *Vibrio* spp. isolated from freshwater (628) and brackish water (342) samples that were confirmed by PCR was 79% (497/628) and 85% (291/342), respectively. Twenty-two percent of the PCR-confirmed *Vibrio* isolates from freshwater (*n* = 497) samples and 41% of the PCR-confirmed *Vibrio* isolates from brackish water samples (*n* = 291) fall among the *Vibrio* species of interest. The incidences of *V. cholerae*, *V. mimicus*, *V. fluvialis*, *V. vulnificus*, *V. alginolyticus*, and *V. parahaemolyticus* amidst these *Vibrio* spp. of interest that were recovered from freshwater samples were 75, 14, 4, 6, 1, and 1%, whereas those from brackish water samples were 24, 7, 3, 3, 47, and 18%, respectively. Our observation during the study suggests pollution as the reason for the unusual isolation of medically important vibrios in winter. Correlation analysis revealed that temperature drives the frequency of isolation, whereas salinity drives the composition of the targeted *Vibrio* species at our sampling sites. The finding of the study is of public health importance going by the usefulness of the water resources investigated. Although controlling and preventing most of the factors that contribute to the prevalence of medically important bacteria, such as *Vibrio* species, at the sampling points might be difficult, regular monitoring for creating health risk awareness will go a long way to prevent possible *Vibrio*-related infection outbreaks at the sampling sites and their immediate environment.

## Introduction

The *Vibrio* genus is made up of over one hundred species (Romalde et al., 2014; Fernández-delgado et al., 2015) of which about 12 have been associated with human infections. The three major human pathogens in the *Vibrio* genus are *Vibrio cholerae*, *Vibrio vulnificus*, and *Vibrio parahaemolyticus* (Guardiola-avila et al., 2015; Kokashvili et al., 2015; Jones, 2017). Pathogenic members of the *Vibrio* genus are very common in the aquatic environment and can cause water- and food-related infections (Ajin et al., 2016; Kirchberger et al., 2016; Machado and Bordalo, 2016; Shaheen et al., 2016). Infections caused by these human pathogenic *Vibrio* species include cholera and vibriosis, e.g., wound infections, septicemia, and gastroenteritis, which is often self-limiting (Baker-Austin et al., 2018). Human pathogenic *Vibrio* species include *V. carchariae*, *V. mimicus*, *V. cincinnatiensis*, *V. fluvialis*, *V. cholerae*, *V. parahaemolyticus*, *V. vulnificus*, *V. alginolyticus* (now *Grimontia hollisae*), *V. furnissii*, *V. metschnikovii*, *V. hollisae*, and *V. damsela* (*Photobacterium hollisae*; Finkelstein, 1996; Levinson and Jawetz, 1996; Turner, 1997; Morris, 2019). The most notable human *Vibrio* pathogens are *V. cholerae*, *V. parahaemolyticus*, *V. vulnificus*, and *V. fluvialis* (CDC, 1999; Finkelstein et al., 2002; Kothary et al., 2003; Chakraborty et al., 2006). On the other hand, *V. mimicus*, which diverged from a common ancestor as *V. cholerae*, and *V. alginolyticus*, which was formerly classified as *V. parahaemolyticus*, are emerging human pathogens (Mustapha et al., 2013; Guardiola-avila et al., 2015). *V. cholerae* causes cholera, whereas other human pathogenic members of the *Vibrio* genus cause infections generally refer to as vibriosis (Baker-Austin et al., 2018). The vehicles of transmission of the etiological agents of cholera and vibriosis to humans are water and food most especially seafood (Di et al., 2017). Water- and food-related diseases continue to be a huge problem for humanity (Tantillo et al., 2004). To circumvent the scourge of cholera and vibriosis, localized monitoring of the environment for etiological agents of the infections was recommended, and this should be done without prejudice to either *Vibrio*-related outbreak is ongoing or not (CDC, 2015). To better protect human health, monitoring the environment for agents of waterborne and foodborne infections, such as human pathogenic *Vibrio* spp., has been emphasized in the literature (European Commission, 2001; Baffone et al., 2006; Jones and Oliver, 2009). Members of the *Vibrio* genus are halophiles; thus, they are not expected to be found in freshwater resources, and this may explain why studies on the prevalence of human pathogenic *Vibrio* spp. in freshwater are relatively small. However, the ability of *Vibrio* spp. to adapt to varying ecological niches (Ceccarelli and Colwell, 2014; Payne et al., 2016; Osunla and Okoh, 2017) supported the need to investigate the role of freshwater resources in the spread of etiological agents of cholera and vibriosis.

Of the approximately 139 serogroups of *V. cholerae*, only O1 and O139 cause pandemic and epidemic cholera, but the non-O1/non-O139 serogroups also cause sporadic cholera-like infections (Glenn Morris, 1994; Rippey, 1994; Sharma et al., 1998; Deen et al., 2019). On the other hand, *V. parahaemolyticus* causes most of the seafood-related diarrhea infections in Florida (Klontz et al., 1993; Letchumanan et al., 2015), and it causes septicemia occasionally (Rippey, 1994; Letchumanan et al., 2015). *V. vulnificus* biotype 1 is the most deadly of all *Vibrio* species because of its high invasiveness and its fatality rate that is higher than that of any other bacteria (Oliver et al., 1991; Oliver and Bockian, 1995; Bisharat, 2002; Bisharat et al., 2005; Drake et al., 2007). *V. fluvialis* commonly cause food poisoning (Bhattacharjee et al., 2010; Kanungo et al., 2012; Chowdhury et al., 2013), and it is an emerging pathogen that possesses epidemic potentials (Vinothkumar et al., 2013). Cholera and vibriosis outbreaks had been reported from several parts of the globe. The isolation of these pathogens from various aquatic milieu around the globe (Sarkar et al., 1985; Venkateswaran et al., 1989; Barua and Greenough, 1991; Kazi et al., 2005; Binh et al., 2009; Safa et al., 2009; Grim et al., 2010; Reimer et al., 2011; Esteves et al., 2015; Okoh et al., 2015; Di et al., 2017; Abioye and Okoh, 2018) has been reported. The occurrence of cholera in the Eastern Cape Province (ECP) has been well documented over the years (Mudzanani et al., 2003; Mema, 2008). The previous studies carried out in our laboratory (Applied and Environmental Microbiology Research Group, Department of Biochemistry and Microbiology, University of Fort Hare, South Africa) confirmed the occurrence of medically important *Vibrio* species in some wastewater treatment plants (WWTPs) of the ECP and their receiving watershed, especially those that discharge poorly treated final effluent (Igbinosa et al., 2009, 2011a,b; Nongogo and Okoh, 2014; Okoh et al., 2015).

These previous findings support the need for spatial and temporal investigation of the presence and distribution of these bacterial species in freshwater resources in the province since there is interconnectivity between WWTPs receiving watershed and other freshwater resources most especially rivers. Going by the scarcity of freshwater resources in South Africa, this kind of investigation is needful since all available freshwater resources in the country are potential alternatives to regular sources of treated freshwater during freshwater scarcity. Some of the anthropogenic activities that contribute to the occurrence of pathogenic *Vibrio* spp. in the aquatic environment include indiscriminate dumping of refuses into water bodies, discharge of poorly treated wastewater from the treatment plant into receiving watershed, run-off from farms especially farmland treated with manure, and defecating and urinating into water bodies by humans and grazing animals (Nevondo and Cloete, 2001; USEPA, 2001; Rees et al., 2010; Abakpa et al., 2013). Contamination due to the aforementioned factors is evident around some of the freshwater resources in the ECP. This is not a surprise since most of the ECP waterbodies are not well protected (Christine et al., 2013). Diseases outbreak in Eastern Cape as a result of watershed contaminated with poorly treated effluent from WWTPs has occurred in the past (Cottle and Deedat, 2002). Unfortunately, recent studies showed that the problem persists in the ECP (Mema, 2008; Nongogo and Okoh, 2014; Okoh et al., 2015; Edokpayi et al., 2017). Poorly treated WWTP’s effluent has been identified as a significant contributor of pathogens to the water milieu of the ECP (Okeyo et al., 2018).

*Vibrio* spp. of medical importance including four of the six *Vibrio* spp. focused on in this study have been reported from various WWTPs of Eastern Cape and their receiving watershed. A significant amount of *V. cholerae*, *V. parahaemolyticus*, *V. metschnikovii*, *V. fluvialis*, and *V. vulnificus* were reportedly isolated from final effluents in some WWTPs in Nkonkobe rural community, Chris Hani, and Amathole district municipalities (Igbinosa et al., 2009, 2011a,b; Nongogo and Okoh, 2014). A more comprehensive study on final effluents of 14 WWTPs in Amathole and Chris Hani district municipalities reported 66.8% *Vibrio* spp. prevalence of the 1,000 randomly selected isolates recovered from the WWTPs. Of the 300 confirmed *Vibrio* spp., 68.2% belong to one of *V. parahaemolyticus*, *V. fluvialis*, and *V. vulnificus* (Okoh et al., 2015). The aforementioned is of potential health risk to individuals using watershed for recreational, agricultural, and domestic purposes most especially at the downstream of WWTP final effluents discharge points. In some studies from other provinces of South Africa, the isolation of *Vibrio harveyi*, *V. parahaemolyticus*, *V. cholerae*, *V. mimicus*, and *V. vulnificus* from tap, borehole, and dam in North West province (Maje et al., 2020) and *V. cholerae* from four WWTPs located in Gauteng Province (Dungeni et al., 2010) has also been reported. Health risks that an individual using these water resources could be exposed to include gastrointestinal infections caused by *V. parahaemolyticus*, *V. mimicus*, and *V. fluvialis*; wound infections caused by *V. vulnificus* and *V. harveyi*; cholera caused by *V. cholerae*; and cholera-like bloody diarrhea caused by *V. fluvialis* (Ramamurthy et al., 1994; Igbinosa and Okoh, 2010; Jones, 2017; Brehm et al., 2020).

The occurrences of dysfunctional WWTPs that discharge unacceptable final effluent (Herbig and Meissner, 2019) and the lack of adequate protection for surface water in the ECP suggest that pathogenic vibrios could have contaminated some water resources in the province. Although extensive work has been done on the contribution of WWTPs to the abundance of *Vibrio* spp. in the province, however, work on major rivers, their tributaries, and brackish water resources is still limited.

The findings of Momba et al. (2006), which confirmed the presence of toxigenic *V. cholerae* in the surface and groundwater of rural locality of Nkonkobe Local Municipality of Amathole District, Eastern Cape Province, South Africa, showed that surface water is of significant importance to the understanding of the abundance of *Vibrio* spp. in the province water milieu. However, the study by Momba et al. (2006) reported for toxigenic *V. cholerae* alone but not for non-cholera causing *Vibrio* pathogens of public health importance. In addition, the study was carried out more than a decade ago, and it does not cover all the important surface water resources in the province. The only one report found on the occurrence of *V. vulnificus* while developing this manuscript was from the KwaZulu-Natal beach, but the information was found in local news (Comins, 2012) rather than in research articles publishing outlets. The other few reports on occurrences of cholera and non-cholera causing *Vibrio* pathogens in rivers were carried out in KwaZulu-Natal, Mpumalanga, and Limpopo provinces, South Africa (Bessong et al., 2009; Madoroba and Momba, 2010; Ntema et al., 2014; Marie and Lin, 2018). Although studies on occurrences of cholera and non-cholera causing *Vibrio* pathogens have been carried out in the ECP in the past, they were limited to up, final effluents discharge points, and some meters downstream of the receiving watershed for the WWTPs (Igbinosa et al., 2009, 2011a,b; Nongogo and Okoh, 2014; Okoh et al., 2015). Therefore, our understanding of the abundance and distribution of *Vibrio* spp. beyond the targeted points along the receiving watershed of the WWTPs and rivers that do not serve as receiving watershed for WWTPs in the province is still unclear. Based on the aforementioned, information on the occurrence of the etiological agents of cholera and vibriosis in South Africa freshwater and brackish water resources at the moment is limited most especially in the ECP. Going by the advice of the CDC (2017) to monitor the environment for *Vibrio* pathogens as a means of preventing cholera and vibriosis outbreaks, this study assesses the microbial quality of some important freshwater and brackish water sources in the ECP based on the presence or absence of six medically important *Vibrio* pathogens. The six medically important *Vibrio* spp. targeted were *V. cholerae*, *V. parahaemolyticus*, *V. vulnificus*, *V. fluvialis*, *V. mimicus*, and *V. alginolyticus*. The last two have not been reported from this region before now. It is intended that the study will contribute to the information needed for the epidemiology of the etiological agents of cholera and vibriosis in the aquatic milieu of the province most especially rivers and brackish water resources. It was also anticipated that this study will encourage *Vibrio* pathogens monitoring programs in the brackish and freshwater milieu of the ECP most especially those that humans access regularly.

## Materials and Methods

### Study Areas and Sample Collection

**Figure 1:**
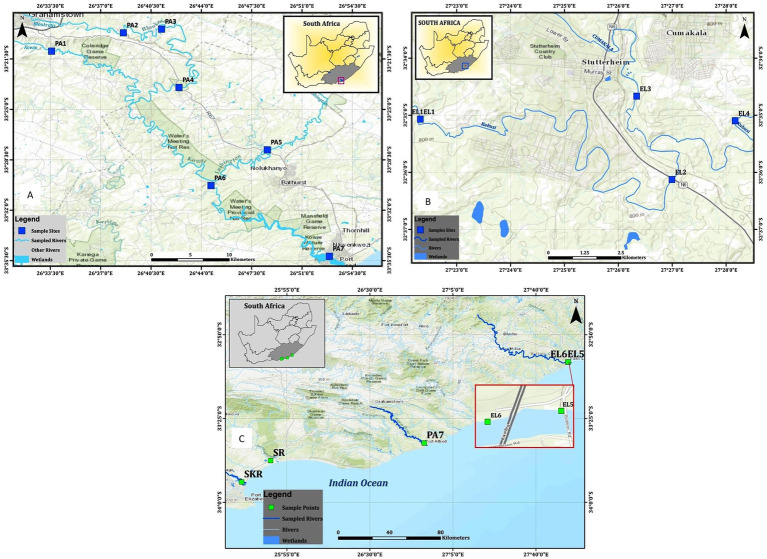
Maps showing locations of the sampling sites, **(A)** sampling sites along the Kowie River, **(B)** sampling sites along the Kubusi River, and **(C)** sampling sites at Buffalo, Sunday, and Swartkops Rivers.

Water samples were collected from freshwater and brackish water resources located along Kowie and tributaries, Kubusi, Sunday, Swartkops, and Buffalo Rivers as shown in Figure 1. Two freshwater dams in Amathole District Municipality were also included in the study, and their coordinates are S32°46.507^l^E026°51.604^l^ and S32°47.406^l^E026°50.821^l^. The dams are used for fishing by the local anglers. The water sampling method employed is as documented in the previous study carried out in our laboratory (Igbinosa et al., 2011b; Okoh et al., 2015). Briefly, water samples were collected at 3–5 different points at each sampling site into 1-L sterile sterilin bottles by midstream dipping of sample bottles at 25–30 cm down the water column. The sample bottle cap was replaced while the sample bottle was still beneath the water column, transferred into cooler boxes containing ice, and afterwards transferred to the Applied and Environmental Microbiology Research Group (AEMREG) laboratory, University of Fort Hare, Alice. All the samples collected were analyzed within 6 h of collection, and samples were collected once a month for 1 year. The characteristics of each of the sampling sites are given in Table 1.

**Table 1:** Sampling site characteristics.

Sample sites/coordinates	Codes	Site peculiarities
Kowie River site 1 (S33°20.953^l^E026°33.601^l^)	PA1	Very close to the source of Kowie River and devoid of any notable human activities during the sampling period. Wild animals have unrestricted access to the water resource at this point.
Bloukrans River site 2 (S33°19.658^l^E026°38.561^l^)	PA2	It is one of the sites along the Kowie River tributary. It is used for agricultural purposes and serves as receiving watershed for wastewater treatment plant.
Bloukrans River site 3 (S33°21.218^l^E026°43.561^l^)	PA3	It is one of the sites along the Kowie River tributary. It is used for agricultural purposes.
Bloukrans River site 4 (S32°23.478^l^E026°42.456^l^)	PA4	It is one of the sites along the Kowie River tributary. It is used for agricultural and spiritual purposes.
Lashinton River site 5 (S32°27.783^l^E026°48.584^l^)	PA5	It is one of the sites along the Kowie River tributary. It is used for agricultural purposes. An awful smell at this site suggests discharge of improperly treated effluent at the upstream.
Kowie River site 6 (S33°30.265^l^E026°44.679^l^)	PA6	Use for fishing activities.
Kowie River Estuary site 7 (S33°35.193^l^E026°53.009^l^)	PA7	It is located in Port Alfred. It is used for recreational activities, e.g., leisure fishing and swimming.
Kubusi River site 8 (S32°35.026^l^E027°22.311^l^)	EL1	Not many human activities occur here, but the upstream is located within a game reserve. It is located at the outskirt of Stutterheim.
Kubusi River site 9 (S32°35.686^l^E027°25.417^l^)	EL2	It is located around one of the local Stutterheim settlements. Free-range cows drink water and defecate at this point. The river is used for agricultural purposes some meters upstream of this site.
Kumukala River 10 (S32°34.675^l^E027°26.350^l^)	EL3	It is one of the tributaries of Kubusi River. It serves as a final effluent receiving watershed for treatment plants. We observed eutrophication and algal bloom at this site. It is very close to Kumukala-Kubusi confluence. Kubusi River is used for an agricultural purpose just immediately after this site.
Kubusi River site 11 (S32°35.078^l^E027°28.168^l^)	EL4	Eutrophication and algal bloom occurred at this site. Some agricultural activities were going on around this site.
Buffalo River Estuary site 12 (S33°01.357^l^E027°53.604^l^)	EL5	An estuarine located along the Buffalo River. It receives industrial effluents, and it is used for fishing activities.
Buffalo River Estuary site 13 (S33°01.385^l^E027°53.470^l^)	EL6	An estuarine located along the Buffalo River. It receives industrial effluents, and it is used for fishing activities. A fish slaughterhouse/cutting plant discharges its untreated effluents at the sampling site.
Sunday River Estuary site 14 (S33°41.580^l^E025°49.563^l^)	SR	An estuary that is located close to Port Elizabeth. Serious fishing and recreational activates occur at this site.
Swartkops River Estuary site 15 (S33°51.444^l^E025°35.940^l^)	SKR	It is an estuary located in Port Elizabeth. Serious fishing and recreational activates occur at this site. Companies and wastewater treatment plants surround the river. The water smells awful, and this suggests that the surrounding companies might be discharging poorly treated effluents into the river.
DAM 1 site 16 (S32°46.507^l^E026°51.604^l^)	ALD1	The dam is used by the University of Fort Hare for agricultural purposes. The presence of cow droppings at the edge of the dam suggests that free-range cows visit the site to drink water. Local fishermen also fish from the dam, especially during the summer season. It is close to Alice wastewater treatment plant.
DAM 2 site 17 (S32°47.406^l^E026°50.821^l^)	ALD2	The dam is used by the University of Fort Hare for agricultural purposes. The presence of cow droppings at the edge of the dam suggests that free-range cows visit the site to drink water. Local fishermen also fish from the dam, especially during the summer season. Tyume, which is the sources of the dam that serves as receiving watershed for a couple of wastewater treatment plants.

### Temperature, Salinity, and Presumptive *Vibrio* Density Determination

The salinity and temperature were determined using a multiparameter ion-specific meter (version HI98195; Hanna Instruments) following the manufacturer’s guidelines. The presumptive *Vibrio* density (PVD) was determined following the membrane filtration technique described in the literature (Nongogo and Okoh, 2014). A 100 ml of raw or diluted water samples, as the case may be, was filtered with the aid of a vacuum pump using a 0.45 μm membrane filter. Afterwards, the resulting membrane filters were placed on sterile thiosulfate citrate bile salts sucrose (TCBS) plates and incubated for 24–48 h at 37°C in triplicates. The mean of the yellow and green colonies on the TCBS triplicate plates per sample was recorded as PVD.

### Total *Vibrio* spp. Density

The most probable number coupled with polymerase chain reaction (MPN-PCR) method detailed in the literature was adapted for determining the total *Vibrio* spp. density (TVD) in water samples (Ahmad et al., 2011; Copin et al., 2012; Ramos et al., 2014; Abioye and Okoh, 2018). As a statistical-based method, MPN only estimates the viable numbers of bacteria in a sample by the principle of extinction dilution, i.e., inoculating liquid medium in 10-fold dilutions to a dilution factor at which no turbidity is observed after the incubation period. Unfortunately, the turbidity observed could be due to the presence of other bacteria other than the organism of interest. To overcome this shortcoming and make the methods more accurate and precise, MPN turbid tubes were subjected to PCR to ascertain the presence of the organism of interest. In this approach, MPN turbid tubes that tested negative for the organism of interest when subjected to PCR are counted as false positive and are not useful for calculating the density. Thus, this approach corrects for the shortcoming of using only MPN for determining the density of specific bacteria species. This is called the MPN-PCR method. Briefly, a 10-fold serial dilution of water sample was prepared up to the power of five, and afterwards, 1 ml of aliquots from raw and each of the diluted water sample was aseptically introduced into test tubes containing 10 ml of sterile freshly prepared alkaline peptone water (APW) in triplicates and incubated at 37°C for 24 h. Turbid tubes were separated, and total genomic DNA was extracted from each turbid tube by the boiling method (Maugeri et al., 2006). For the total genomic DNA extraction, 1 ml of aliquot from the turbid tubes was aseptically transferred into a sterile microcentrifuge tube and centrifuged for 2 min at a speed of 11,000 × *g* using a MiniSpin microcentrifuge. After centrifugation, the supernatant was discarded while 200 μl of sterile distilled water was added to the cell pellet at the bottom of the microcentrifuge tubes and vortexed to form a solution containing evenly distributed cells. The cells in the solution were afterwards lysed with the aid of an AccuBlock (Digital dry bath; Labnet) for 15 min at 100°C, and the solution of the lysed cells was centrifuged at a speed earlier mentioned. The supernatant that was the genomic extraction was subjected to 25 μl PCR reaction to confirm the presence of *Vibrio* spp. using a primer that targets a variable region of *16S rRNA* gene that is specific for members of the *Vibrio* genus. Furthermore, to determine the absolute density of each of the targeted *Vibrio* spp., DNA templates from MPN tubes that were positive for *Vibrio* spp. were subjected to another round of PCR that targeted each of the six *Vibrio* spp. using species-specific primers used for delineation (Table 2). The composition of the PCR reaction was 5 μl of DNA template, 12.5 μl of one Taq 2X Master Mix Standard Buffer (BioLabs, UK), 1 μl each of 10 μM of forward and reverse primers, and 5.5 μl of nuclease-free water. The concentration of the DNA in templates ranges between 80 and 195 ng/μl through the experiment. The positive controls used for the PCR assay were *V. parahaemolyticus* (DSM 10027), *V. vulnificus* (DSM 10143), *V. fluvialis* (DSM 19283), *V. mimicus* (DSM 19130), and *V. alginolyticus* (DSM 2171) and one locally isolated *V. cholera*. The TVD was determined by extrapolating the equivalent MPN values for turbid tubes that were positive for members of the *Vibrio* genus and each of the targeted *Vibrio* spp. using bacteriological analytical manual (BAM) Excel spreadsheet (Blodgett, 2015). The detection limit for the MPN-PCR method is <3 MPN/ml (<0.477 log MPN/ml). The forward sequence for the *Vibrio* genus-specific primer used was 3'CGG TGA AAT GCG TAG AGA T5', whereas that for the reverse sequence was 3'TTA CTA GCG ATT CCG AGT TC5'. The thermal cycler condition for the PCR assay was 15 min at 93°C for initial denaturation, 35 cycles of 92°C for 40 s, 57°C for 1 min, and 72°C for 1.5 min for denaturation, annealing, and elongation, respectively, and 75°C for 7 min for the final extension. The resulting amplicons were electrophoresed on 1.5% agarose, and pictures of amplified DNA bands on agarose gel were viewed and taken using an ultraviolet (UV) transilluminator following the manufacturer’s guideline. The expected amplicon size was 663 bp (Kwok et al., 2002; Okoh et al., 2015).

**Table 2:** List of species-specific primers.

Species	Sequence	Size bp	References
*V. cholerae*	F: 3'-CAC CAA GAA GGT GAC TTT ATT GTG-5' R: 3'-AGG ATA CGG CAC TTG AGT AAG ACTC-5'	304	GOEL et al., 2010; de Menezes et al., 2014
*V. parahaemolyticus*	F: 3'-GCA GCT GAT CAA AAC GTT GAG T-5' R: 3'-ATT ATC GAT CGT GCC ACT CAC-5'	897	Tarr et al., 2007; Okoh et al., 2015
*V. vulnificus*	F: 3'-GTC TTA AAG CGG TTG CTG C-5' R: 3'-CGC TTC AAG TGC TGG TAG AAG-5'	410	Wong and Chow, 2002; Okoh et al., 2015
*V. fluvialis*	F: 3'-GAC CAG GGC TTT GAG GTG GAC GAC-5' R: 3'GGT TTG TCG AAT TAG CTT CAC C-5'	217	Osorio and Klose, 2000; Chakraborty et al., 2006
*V. mimicus*	F: 3'-GGTAGCCATCAGTCTTATCACG-5' R: 3'-ATCGTGTCCCAATACTTCACCG-5'	390	Lei et al., 2000; Shinoda et al., 2004; Sultan et al., 2007; Guardiola-avila et al., 2015
*V. alginolyticus*	F: 3'-GAGAACCCGACAGAAGCGAAG-5' R: 3'-CCTAGTGCGGTGATCAGTGTTG-5'	337	Zhou et al., 2007; Wei et al., 2014

### Isolation of Presumptive *Vibrio* spp. From Water Samples

To maximize the isolation of the *Vibrio* species of interest, a water sample (100 ml) was filtered as articulated in the Temperature, Salinity, and Presumptive *Vibrio* Density Determination section, and the resulting membrane filter was introduced into a conical flask containing sterile 100 ml APW. The conical flask with its content was perturbed gently for about 1–2 min before incubating the set-up for 24 h at 37°C (Ntema et al., 2010). The set-up was observed on hourly bases, and as soon as there was the formation of the pellicle at the surface of the incubated APW, a loopful just below the pellicle was carefully streaked on sterile TCBS (Ceccarelli et al., 2015). The newly streaked TCBS plates were incubated for 24 h at 37°C. At the expiration of the incubation period, colonies (5–10 per plate) with typical *Vibrio* yellowish and greenish morphology were carefully picked, re-streaked on fresh sterile TCBS, and incubated for 24 h at 37°C. After the incubation period, a colony from TCBS plates with uniform colonies’ morphology were transferred to fresh 1% NaCl nutrient agar plates. Resulting colonies were Gram stained and observed under a microscope to ensure the purity of the isolated presumptive *Vibrio* species to be stocked. Twenty percent glycerol stock of pure isolates were prepared afterwards and stored at −80°C for PCR analysis.

### Molecular Identification of Presumptive *Vibrio* Isolates

A total genomic DNA was extracted from the presumptive *Vibrio* isolates using the boiling method as described earlier in the Total *Vibrio* spp. Density section. The DNA was extracted from a colony of an 18-h-old pure culture of presumptive *Vibrio* spp. isolates. A solution of the colony prepared in a microcentrifuge containing 200 μl of sterile distilled water was boiled, and total genomic DNA was afterwards extracted from the cell solutions as earlier described in the Total *Vibrio* spp. Density section. This was followed by the confirmation of all the presumptive isolates as member of the *Vibrio* genus or otherwise following the PCR protocol detailed in the Total *Vibrio* spp. Density section. All PCR-confirmed *Vibrio* isolates were further delineated into the six *Vibrio* spp. targeted in this study.

### Delineation of the PCR-Confirmed *Vibrio* Species

A set of species-specific primers were employed in PCR assay for delineating confirmed *Vibrio* spp. into the *Vibrio* species of interest. The respective primers employed for the identification of *V. cholerae*, *V. mimicus*, *V. fluvialis*, *V. vulnificus*, *V. alginolyticus*, and *V. parahaemolyticus* targeted the conserved region on *OmpW*, *vhm*, *ToxR*, *GroEl*, *GyrB*, and *fla E* genes that are specific for the organisms of our interest. *V. cholera* and *V. mimicus* were simultaneously identified using a duplex PCR protocol, whereas *V. vulnificus*, *V. fluvialis*, and *V. alginolyticus* were also simultaneously identified by employing a triplex PCR protocol. *V. parahaemolyticus* isolates were identified using a simplex PCR protocol. The primer sequences, sources, and expected amplicon sizes are given in Table 2, and all the PCR protocols were as earlier articulated in our previous study (Abioye and Okoh, 2018). The resulting amplicons were electrophoresed, bands on gels were viewed, and their pictures were taken using a UV transilluminator following the manufacturer’s guideline.

### Statistical Analysis

Results for freshwater and brackish water samples were distinctively treated on seasonal and annual bases. Density across sites was statistically compared using the Kruskal-Wallis test and the Games-Howell *post hoc* test at *p* ≤ 0.05. Spearman correlation analysis was carried out to understand the relationship between PVD, TVD, temperature, and salinity at our sampling sites since salinity and temperature have been reported to modulate the ecology of *Vibrio* spp. differently at different geographical locations. The frequency of isolation (relative density) and absolute density of each of the six targeted *Vibrio* spp. were also correlated with temperature and salinity to establish species-based relationship. Kruskal-Wallis test and Spearman correlation analysis were used because our variables (temperature, density, salinity, and frequency of isolation) were not normally distributed.

Excel version 2013 and SPSS version 2020 were used to organize and analyze our data.

## Result and Discussion

### Temperature, Salinity, and *Vibrio* spp. Density

**Figure 2:**
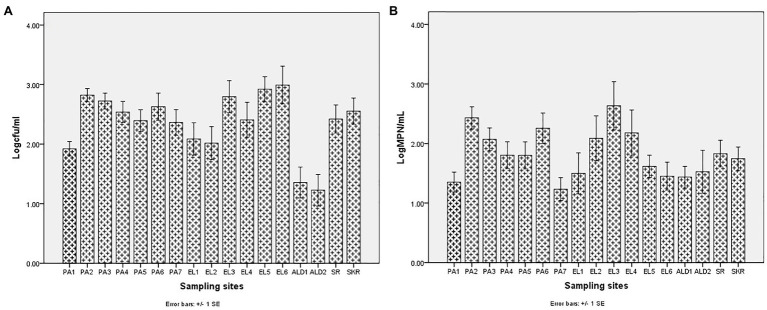
Annual mean density of *Vibrio* spp. **(A)** PVD and **(B)** TVD.

The average annual temperature and salinity of water samples at the sampling points were as given in Table 3. Salinity ranges between 0.06 ± 0.04 PSU at site EL1 and 2.03 ± 0.47 PSU at site PA5 for freshwater sampling sites, whereas it ranges between 16.14 ± 3.91 PSU at site SR and 31.65 ± 4.19 PSU at site PA7 for brackish water sampling sites. The temperature range was between 15.31 ± 4.25°C at site EL1 and 21.50 ± 3.67°C at site PA6 for freshwater sampling sites, and it was between 18.74 ± 2.12°C at site EL6 and 22.01 ± 4.47°C at site SR for brackish water sampling sites. During the study regime, 194 water samples were collected and analyzed for PVD and TVD. The mean annual PVD and TVD per site (Figure 2) differ across the sampling sites. The average annual PVD ranges between 1.23 ± 0.83 log CFU/ml at site ALD2 and 2.92 ± 0.93 log CFU/ml at site EL4 for freshwater samples, whereas that for brackish water samples ranges between 2.36 ± 0.72 log CFU/ml at site PA7 and 2.99 ± 1.0 log CFU/ml at site EL6. On the other hand, the range of annual average TVD was 1.35 ± 0.56 log MPN/ml at site PA1 to 2.63 ± 1.28 log MPN/ml at site EL3 for freshwater samples. The range for brackish water samples was 1.23 ± 0.64 log MPN/ml at site PA7 to 1.83 ± 0.71 log MPN/ml at site SR. The PVD at all sampling sites was more than that at site PA1 except for sites ALD1 and ALD2. This observation was significant at p ≤ 0.05 for all sampling sites except for sites PA7, EL1, EL2, EL4, ALD1, and SR. A similar scenario was observed for TVD except that TVD at site PA1 was greater than TVD at site PA7. The observation for TVD was only significant for PA2 > PA1. The densities of each of the targeted species are as given in Supplementary Table SS1B. A density greater than 0.477 log MPN/ml, which was the maximum detection limit of the three tubes by the five dilutions MPN-PCR density determination method employed, was recorded mainly in summer but scarcely recorded in winter months. Of the samples with >0.477 log MPN/ml *V. cholerae*, *V. mimicus*, *V. fluvialis*, *V. vulnificus*, *V. alginolyticus*, and *V. parahaemolyticus* densities, 82, 72, 63, 100, 87, and 58%, respectively, were collected in summer months.

**Table 3:** Average annual salinity and temperature at the freshwater and brackish water sampling sites.

Sites	Salinity ± SD (PSU)	Temperature ± SD (°C)
**Freshwater sampling sites**		
PA1	0.18 ± 0.04	18.40 ± 3.14
PA2	0.53 ± 0.17	18.02 ± 3.00
PA3	0.69 ± 0.13	18.30 ± 3.11
PA4	0.71 ± 0.11	19.12 ± 3.17
PA5	2.03 ± 0.47	19.67 ± 3.41
PA6	1.70 ± 0.60	21.50 ± 3.67
EL1	0.06 ± 0.04	15.31 ± 4.24
EL2	0.11 ± 0.09	15.61 ± 4.30
EL3	0.13 ± 0.05	16.45 ± 3.80
EL4	0.14 ± 0.19	16.40 ± 4.18
ALD1	0.12 ± 0.07	18.81 ± 5.47
ALD2	0.13 ± 0.12	19.77 ± 4.68
**Brackish water sampling sites**		
PA7	31.65 ± 4.19	20.10 ± 2.07
EL5	29.14 ± 8.69	19.37 ± 1.46
EL6	27.94 ± 8.84	18.74 ± 2.12
SR	16.14 ± 3.91	22.10 ± 4.47
SKR	28.45 ± 2.29	21.63 ± 3.56

Key: SD = standard deviation

**Figure 3:**
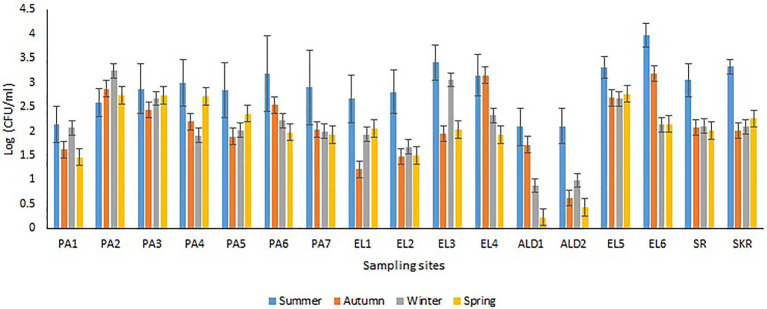
Seasonal variations in PVD.

**Figure 4:**
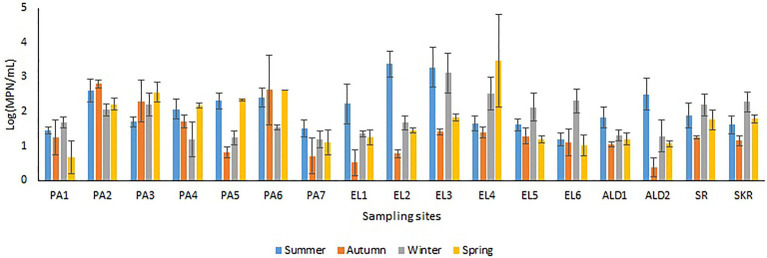
Seasonal variations in TVD.

Sites PA1, EL1, ALD1, and ALD2 were anticipated to be pristine and have relatively low microbial load because PA1 and EL1 were around the sources of Kowie and Kubusi Rivers, respectively, whereas sites ALD1 and ALD2 were in a controlled environment. However, in some instance, PVD and TVD at these supposedly said pristine sampling sites were greater than PVD and TVD at other sampling sites, e.g., the mean annual *Vibrio* density at PA1 was greater than that at PA7, which are more exposed to anthropogenic activities. The unanticipated observation could be as a result of the easy access and activities of wild and domesticated animals around the water resources at PA1. South Africa water resources have been adjudged unprotected (Christine et al., 2013; Colvin et al., 2016) while domesticated and wild animals activities around surface water generally cause contamination of water resources with pathogens (Cabral, 2010; McAllister and Topp, 2012; Decol et al., 2017; Topalcengiz et al., 2017; Liu et al., 2018). Furthermore, domestic wastes from informal settlements along the riverbanks, effluents from overloaded and poorly maintained treatment plants, polluted runoff, and solid wastes that are potential contributors of pathogens to surface water (Fatoki et al., 2001; Chigor et al., 2012; Edokpayi et al., 2017; Adams et al., 2019) were evident at our sampling sites. In addition, droppings from free-range and grazing cows were observed at most of our freshwater sampling sites. Animals droppings are major contributors of pathogenic *Vibrio* spp., e.g., toxigenic *V. cholerae* into human environments (Dufour et al., 2013; Momba and Azab El-Liethy, 2017; Penakalapati et al., 2017; Delahoy et al., 2018; Munshi et al., 2019). As anticipated, sites SR, SKR, PA7, EL5, and EL6 located at estuaries where *Vibrio* spp. are commonly isolated and site EL6 that also serves as receiving watershed for fish slaughterhouse/cutting plant had relatively high *Vibrio* density than some of the freshwater sampling sites. Reports have shown that effluents from fish slaughterhouses contribute significantly to *Vibrio* spp. load in receiving watershed (Novotny et al., 2004; Skall and Olesen, 2011). Surprisingly, the densities of *Vibrio* at some of the freshwater sampling sites were also significantly more than those at some of the brackish water sampling sites (Figures 2A,B). This observation could be as a result of the type of industrial effluent and other pollutants that are usually discharged into the water environments around the brackish water sampling sites (Adeniji et al., 2017a,b). Industrial waste has been reported to modulate the microbial biodiversity of the aquatic ecosystem (Li et al., 2017; Jordaan et al., 2019), and this could be on a downward trend for microbial populations when such waste is toxic rather than nutrient-rich. *Vibrio* spp. density (PVD and TVD) also varies across seasons with relatively high density recorded in summer as earlier reported (Igbinosa et al., 2011a,b; Kokashvili et al., 2015); however, few exceptions were observed (Figures 3, 4). A relatively high PVD and TVD in winter recorded at sites PA2 and EL3 can be traced to pollution. The two sites serve as receiving watershed for dysfunctional WWTPs (Maclennan, 2019), and dysfunctional WWTPs usually contaminate the water system with pathogens (Edokpayi et al., 2017). The highest density for each of the targeted *Vibrio* spp. (Supplementary Table SS1B) was recorded at sites (PA2, PA5, EL3, SR, and SKR) where we observed a high level of pollution.

Statistically speaking, a significantly positive weak correlation (*r* = 0.201, *p* = 0.018) between PVD and salinity was observed for freshwater but a significantly negative weak correlation (*r* = −0.327, *p* = 0.014) for brackish water samples. The correlation between TVD and salinity was not significant for freshwater (*r* = 0.058, *p* = 0.503) and brackish water (*r* = −0.163, *p* = 0.203) samples. The correlation coefficients between PVD vs. temperature (*r* = 0.133, *p* = 0.121 for freshwater; *r* = 0.2, *p* = 0.14 for brackish water) and TVD vs. temperature (*r* = 0.018, *p* = 0.252 for freshwater; *r* = −0.068, *p* = 0.619 for brackish water) were not significant too. The lack of correlation between salinity, temperature, and *Vibrio* density had been reported in the literature (Randa et al., 2004; De Souza Costa Sobrinho et al., 2010; Paranjpye et al., 2015; Nilsson et al., 2019). The finding suggests that the contribution of salinity and temperature to the *Vibrio* genus density at our sampling sites is small, and that some other physicochemical and biological factors could be key to *Vibrio* density dynamics at our sampling sites. Interestingly, the literature has shown that parameters, such as dissolved oxygen and chemical and biological oxygen demands, have a strong relationship with *Vibrio* spp. density and the aforementioned parameters are good indexes for detecting water resources polluted with organic waste that may contain pathogens (Sharma and Chaturvedi, 2007; Prasanthan et al., 2011; Mezgebe et al., 2015; Di et al., 2017; Rabea et al., 2019). Unfortunately, organic waste pollution can occur at any time and cause unexpected offshoot in microbial density and diversity in an ecological niche. This, thus, explain the observation of the highest *Vibrio* density in seasons other than summer at some of our sampling sites because we observed that sources of organic waste pollutants are close to these sampling sites.

### Effects of Salinity and Temperature on the Density, Frequency of Isolation, and Seasonality of the Targeted *Vibrio* Species

**Figure 5:**
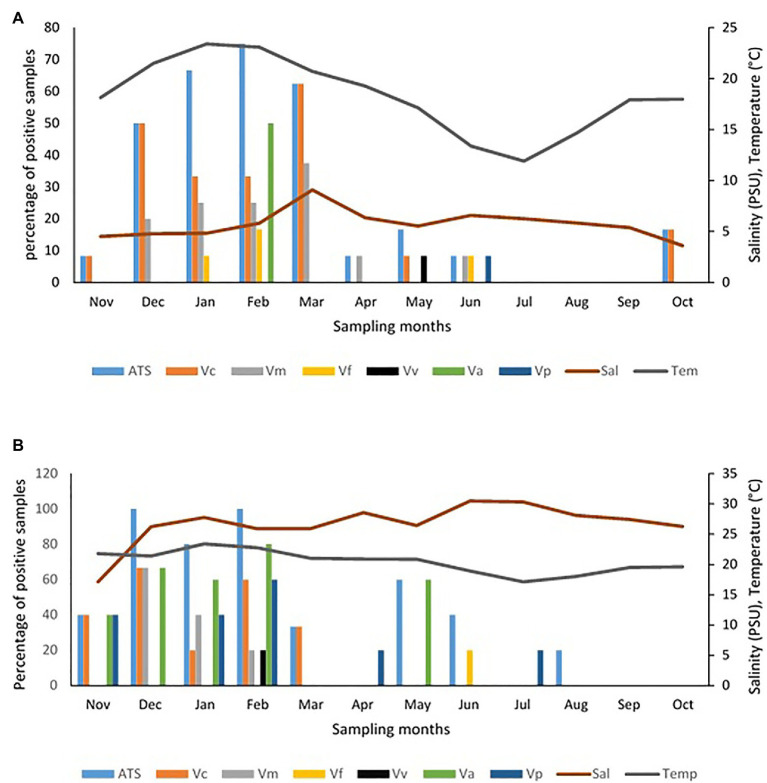
Influence of temperature and salinity on the detection of targeted *Vibrio* spp. in water samples. **(A)** Freshwater samples, **(B)** brackish water samples. Key: Va = *V. alginolyticus*, Vc = *V. cholerae*, Vf = *V. fluvialis*, Vm = *V. mimicus*, Vp = *V. parahaemolyticus*, Vv = *V. vulnificus*, ATS = all targeted *Vibrio* species.

**Figure 6:**
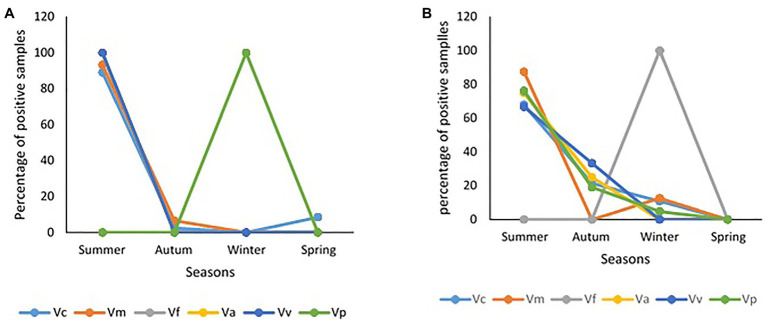
Seasonality of samples positive for targeted *Vibrio* spp. **(A)** freshwater samples, **(B)** brackish water samples.

The frequency of isolation of each of the *Vibrio* species of interest in freshwater and brackish water samples per sampling month and the impact of temperature and salinity on the frequency of detection are given Figure 5. The seasonal variation in the frequency of isolation of the *Vibrio* spp. of interest is given in Figure 6. The results show that the organism of interest was frequently isolated in summer than other seasons. Surprisingly, while the isolation frequency of organisms of interest in freshwater samples followed a similar pattern as salinity and temperature, only temperature did for brackish water samples. It is an established fact that salinity and temperature are important environmental factors that influence *Vibrio* spp. density, but the literature has also shown that the correlation between these factors and *Vibrio* spp. density differs for different geographical locations and sample types (Kelly, 1982; Kaspar and Tamplin, 1993; Fukushima and Seki, 2004; Randa et al., 2004; Igbinosa et al., 2011a; Takemura et al., 2014; Kokashvili et al., 2015; Colwell, 2016; Urquhart et al., 2016). For example, Kokashvili et al. (2015) observed a linear correlation between the two factors and frequency of isolation of some medically important *Vibrio* species but a negative correlation between temperature and *V. metschnikovii* at the marine and freshwater sampling sites. In addition, Randa et al. (2004) observed a significant moderate negative correlation between salinity and abundance of *V. vulnificus* in estuarine water samples, whereas Colwell (2016) reported a non-significant negative correlation between temperature and the abundances of *V. parahaemolyticus* and *V. vulnificus* in estuary water samples. In this study, the frequency of isolation of at least one of the targeted species from freshwater samples showed a significant positive relationship with salinity and temperature (Table 4). However, only temperature had a significant positive relationship with the frequency of isolation of at least one of the targeted *Vibrio* spp. in brackish water samples. Interestingly, correlation coefficients and *p* values for the relationship between frequency of isolation of at least one of the *Vibrio* spp. and temperature were very similar for the two water types (Table 4). This shows that temperature modulates the chances of isolating at least one of the *Vibrio* spp. of interest from the two water types in a similar way. However, at species level, the correlation analysis (Table 4) showed that temperature is more effective as guide for the isolation of *V. cholerae*, *V. mimicus*, and *V. fluvialis* (generally called non-halophilic) from freshwater and *V. alginolyticus*, *V. vulnificus*, and *V. parahaemolyticus* (generally called halophilic) from brackish water. In their work, Liu et al. (2016) and Mahoney et al. (2010) reported that temperature is more relevant to the isolation of non-halophilic vibrios from freshwater and halophilic vibrios from brackish water than salinity. Consequently, we adjudged from our data (Figure 5) that a temperature range between 20 and 24°C is the optimum temperature for the isolation of *V. cholerae*, *V. mimicus*, and *V. fluvialis* from freshwater sampling sites and *V. alginolyticus*, *V. vulnificus*, and *V. parahaemolyticus* from brackish water sampling sites. It was further inferred that a salinity range between 5 and 9 PSU should be used in adjunct with a temperature range of 20 and 24°C for the isolation of *V. cholerae* at the freshwater sampling sites. Our submission is in concordance with earlier studies that showed that relatively high frequency of isolation of medically important *Vibrio* spp. is usually achieved in summer when the temperature is above 15°C and salinity is between 5 and 25 PSU (Jones and Summer-Brason, 1998; DePaola et al., 2003; Urquhart et al., 2016).

**Table 4:** Correlation analysis between the frequency of isolation of targeted *Vibrio* spp. from water samples, temperature, and salinity.

Freshwater				Brackish water			
Organism type	Parameters	R	*p*	Organism type	Parameters	R	*p*
FITV	Sal	**0.215**	**0.011**	FITV	Sal	0.094	0.497
	Temp	**0.462**	**<0.0001**		Temp	**0.459**	**<0.0001**
FIVc	Sal	**0.33**	**<0.0001**	FIVc	Sal	0.193	0.153
	Temp	**0.411**	**<0.0001**		Temp	0.139	0.308
FIVm	Sal	0.028	0.747	FIVm	Sal	−0.010	0.941
	Temp	**0.192**	**0.023**		Temp	0.072	0.596
FIVf	Sal	−0.1	0.244	FIVf	Sal	0.121	0.374
	Temp	**0.234**	**0.006**		Temp	−0.140	0.305
FIVa	Sal	−0.022	0.801	FIVa	Sal	0.03	0.828
	Temp	0.153	0.073		Temp	**0.51**	**<0.0001**
FIVv	Sal	−0.055	0.521	FIVv	Sal	−0.233	0.084
	Temp	−0.064	0.453		Temp	**0.351**	**0.008**
FIVp	Sal	−0.061	0.477	FIVp	Sal	−0.057	0.679
	Temp	−0.067	0.435		Temp	**0.571**	**<0.0001**

Key: Bolded R and *p* values show a significant correlation. FIA = frequency of isolation; TV = the targeted *Vibrio* spp.; Va = *V. alginolyticus*; Vc = *V. cholerae*; Vf = *V. fluvialis*; Vm = *V. mimicus*; Vp = *V. parahaemolyticus*; Vv = *V. vulnificus*; %P = percentage prevalence

It is good to point out that targeted *Vibrio* spp. from few samples at sites ALD1, ALD2, EL5, and SKR were isolated in winter in this study. This could be attributed to the level of pollution at these sites as earlier discussed and affirm the possibility of isolating medically important *Vibrio* spp. in usual season because of pollution and contamination that is not season dependent. It has been earlier reported by Kopprio et al. (2020) and Watkins and Cabelli (1985) that warmer temperatures and sewage pollution are reasons for the abundance of pathogenic *Vibrio* spp. in the aquatic milieu. In addition, the studies carried out by Jaiani et al. (2013) and Janelidze et al. (2011) suggest that temperature affects *Vibrio* abundance, whereas salinity affects *Vibrio* species composition of any ecological niche. It is important to note here that a similar scenario discussed above was observed when absolute densities of targeted *Vibrio* spp. were statistically analyzed (Supplementary Tables SS1B,C; Supplementary Figures SS1A,B).

### Prevalence of the Six Targeted *Vibrio* Species of Medical Importance

**Figure 7:**
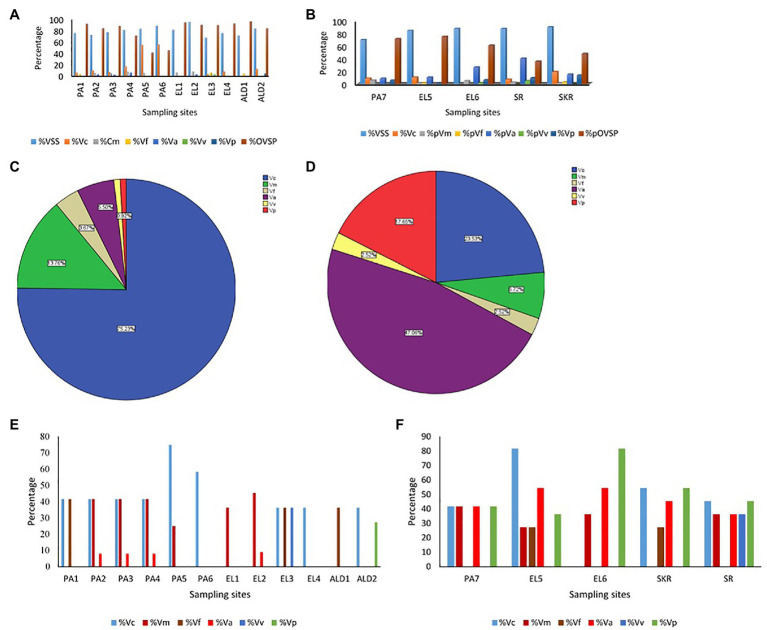
**(A,B)** Prevalence of targeted *Vibrio* spp. in isolates recovered per sampling sites. **(C,D)** Distribution of targeted *Vibrio* spp. among isolates recovered from samples. **(E,F)** Detection rate of targeted *Vibrio* spp. in samples per sampling site. **(A,E)** Freshwater sampling sites, **(B,F)** brackish water sampling sites, **(C)** isolates from freshwater samples, **(D)** isolates from brackish water samples, % = percentage, VSS = *Vibrio* spp. positive samples, Vc = *Vibrio cholerae*, Vm = *Vibrio mimicus*, Vf = *Vibrio fluvialis*, Va = *Vibrio alginolyticus*, Vp = *V. parahaemolyticus*, OVSP = other *Vibrio* spp.

**Figure 8:**
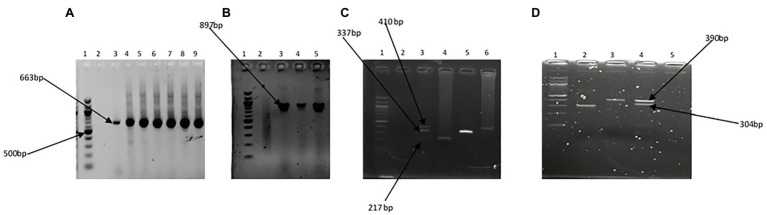
Gel pictures showing PCR amplification products of the specific regions of *16S rRNA* gene for the *Vibrio* genus **(A)**, *fla E* gene for *V. parahaemolyticus* **(B)**, *GroEl*, *ToxR*, and *GyrB* genes in triplex PCR for *V. vulnificus*, *V. fluvialis*, and *V. alginolyticus*, respectively **(C)**, and *OmpW* and *vhm* genes in duplex PCR for *V. cholerae* and *V. mimicus*, respectively. Lane 1 **(A–D)** 100 bp molecular marker, lane 2 **(A–C)** and lane 5 **(D)** negative control, lane 3 **(A–C)** and lane 4 (D) positive controls, and lanes 4–9 **(A)**, lanes 4 and 5 **(B)**, lanes 4–6 **(C)**, and lanes 2 and 3 **(D)** positive isolates.

The Kruskal-Wallis test showed that the average annual absolute density of each of the targeted *Vibrio* spp. was significantly different across sites at *p* < 0.05. The Games-Howell *post hoc* test showed the site comparisons with significant differences (Supplementary Table SS1D). The comparisons of absolute density of targeted species between freshwater and brackish water sites that showed significant difference revealed that *V. cholerae* and *V. mimicus* are more abundant in freshwater than in brackish water, whereas *V. vulnificus*, *V. alginolyticus*, and *V. parahaemolyticus* are more abundant in brackish water than in freshwater. Furthermore, seasonal comparison of absolute density (Supplementary Table SS1E) showed that all the targeted species proliferate in summer significantly than in other seasons of the year. The relative abundance of each of the six targeted *Vibrio* spp. in isolates recovered at the sampling sites is shown in Figures 7A,B, whereas the incidence of each of the targeted species among the total *Vibrio* spp. isolates recovered from each of the water sample types is given in Figures 7C,D. The prevalence of each of the targeted *Vibrio* spp. per sampling site is given in Figures 7E,F. At least one of the targeted *Vibrio* species was detected and isolated from each of the sampling sites. The prevalence of *V. cholerae*, *V. mimicus*, *V. fluvialis*, *V. vulnificus*, *V. alginolyticus*, and *V. parahaemolyticus* in freshwater samples was 34, 19, 9, 2, 3, and 2%, respectively. On the other hand, the prevalence of *V. cholerae*, *V. mimicus*, *V. fluvialis*, *V. vulnificus*, *V. alginolyticus*, and *V. parahaemolyticus* in brackish water samples was 44, 28, 10, 7, 46, and 51%, respectively. A total of 628 and 342 presumptive isolates from freshwater and brackish water samples, respectively, were analyzed in this study. Of the freshwater presumptive isolates, 79% were confirmed as *Vibrio* spp., whereas 85% of presumptive isolates from brackish water samples were confirmed as *Vibrio* spp. Twenty-two and 41% of the PCR-confirmed isolates from freshwater samples and brackish water samples, respectively, fall among the targeted *Vibrio* species. The samples of gel electrophoresis pictures for the confirmation and speciation of *Vibrio* spp. into the six targeted species are given in Figure 8. The incidence of *V. cholerae*, *V. mimicus*, *V. fluvialis*, *V. vulnificus*, *V. alginolyticus*, and *V. parahaemolyticus* of all the six targeted *Vibrio* species recovered from freshwater samples was 75, 14, 4, 6, 1, and 1%, whereas that for brackish water was 24, 7, 3, 47, 3, and 18%, respectively. The predominant species of the freshwater samples were *V. cholerae* and *V. mimicus*, whereas those of the brackish water samples were *V. alginolyticus*, *V. cholerae*, and *V. parahaemolyticus*. The prevalence of the remaining four (*V. fluvialis*, *V. vulnificus*, *V. alginolyticus*, and *V. parahaemolyticus*) of the six targeted *Vibrio* spp. among *Vibrio* isolates from freshwater samples and three (*V. mimicus*, *V. fluvialis*, *V. vulnificus*) of the six targeted *Vibrio* species in brackish water samples were, respectively, low. Our result showed population diversity of targeted *Vibrio* species in freshwater and brackish water samples. Halophilic vibrios were more abundant in brackish water, whereas non-halophilic vibrios were abundant in freshwater samples. The six targeted *Vibrio* spp. were more diverse in brackish water sample than in freshwater sample. These findings corroborate some earlier reports and suggest that salt concentration modulates the diversity of vibrios in water resources (Elhadi, 2013; Mookerjee et al., 2015; de Menezes et al., 2017; Fu et al., 2019). The result (Figure 7; Supplementary Table SS1A) of the present study showed that the possibility of contracting infections, such as cholera and cholera-like infections, caused by the non-halophilic vibrios is higher at the freshwater sampling sites most especially sites PA4, PA5, PA6, and ALD1 where most of the halophilic vibrios were recovered than at the brackish water sampling sites. Likewise, the chances of contracting gastroenteritis, wound infections, and other vibriosis peculiar to halophilic vibrios are higher at the brackish sampling sites most especially sites EL6, SR, and SKR. Furthermore, the coexistence of medically important non-halophilic and halophilic vibrios at the same ecological niche in most of our sampling sites is of public health concern. As the density and diversity of cholera and vibriosis causing *Vibrio* species increase in a single ecological niche, the chances of acquiring the infections they cause also increase. Furthermore, the possibility of exchanging genetic materials, such as virulence and antibiotics resistance determinants, will be high. The coexistence of medically important *Vibrio* species within the same ecological niche has been reported in an earlier study (Kokashvili et al., 2015). Although the present study focused on freshwater and brackish water that have not been investigated for members of the *Vibrio* genus before, *Vibrio* spp. of medical importance including four of the six *Vibrio* spp. focused on in this study have been reported from various WWTPs of Eastern Cape and their receiving watershed. A significant amount of *V. cholerae*, *V. parahaemolyticus*, *V. metschnikovii*, *V. fluvialis*, and *V. vulnificus* were reportedly isolated from final effluents in some WWTPs in Nkonkobe rural community, Chris Hani, and Amathole district municipalities (Igbinosa et al., 2009, 2011a,b; Nongogo and Okoh, 2014). A more comprehensive study on final effluents of 14 WWTPs in Amathole and Chris Hani district municipalities reported 66.8% *Vibrio* spp. prevalence of the 1,000 randomly selected isolates recovered from the WWTPs. Of the 300 confirmed *Vibrio* spp., 68.2% belong to one of *V. parahaemolyticus*, *V. fluvialis*, and *V. vulnificus* (Okoh et al., 2015). All these studies, including the present one, show that medically important *Vibrio* spp. are present in the aquatic milieu of the ECP. The aforementioned is of potential health risk to individuals using the water resources studied and watershed for recreational, agricultural, and domestic purposes most especially at the downstream of WWTP final effluents discharge points. In some studies from other provinces of South Africa, the isolation of *V. harveyi*, *V. parahaemolyticus*, *V. cholerae*, *V. mimicus*, and *V. vulnificus* from tap, borehole, and dam in North West province (Maje et al., 2020) and *V. cholerae* from four WWTPs located in Gauteng Province (Dungeni et al., 2010) has also been reported. Health risks that an individual using these water resources could be exposed to include gastrointestinal infections caused by *V. parahaemolyticus*, *V. mimicus*, and *V. fluvialis*; wound infections caused by *V. vulnificus* and *V. harveyi*; cholera caused by *V. cholerae*; and cholera-like bloody diarrhea caused by *V. fluvialis* (Ramamurthy et al., 1994; Igbinosa and Okoh, 2010; Jones, 2017; Brehm et al., 2020).

## Summary and Conclusion

The present study confirmed the presence of *Vibrio* species of medical importance in both freshwaters (Kowie River, Bloukrans River, Lashinton River, Kubusi River, and two dams in Amathole District Municipality) and brackish water (Buffalo, Sunday, Kowie, and Swartkops estuaries) of the Eastern Cape, Province of South Africa. It is worth mentioning that humans have regular contact with more than 88% of our sampling sites and all the sampling sites had not been investigated for the occurrence of *Vibrio* spp. of medical importance before. The predominant species at the sampling sites showed that the chances of contracting cholera and cholera-like infection are high at the freshwater sampling sites than at the brackish water sampling sites. On the other hand, the chances of contracting other vibriosis are high at the brackish water sampling sites than at the freshwater sampling sites. The findings of the study also suggest pollution as the reason for the isolation of medically important vibrios in the unusual season at some of the sampling sites. It was also observed that temperature drives isolation frequency, whereas salinity drives the composition of the targeted *Vibrio* species at both freshwater and brackish water sampling sites. Although the virulence status of the isolated medically important *Vibrio* species was not elucidated in this study, the confirmation of their presence in the water resources investigated for the first time is a significant finding. This finding is an eye opener to the potential threat that the water resources investigated pose to public health in terms of cholera and vibriosis. Therefore, our findings are of public health importance going by the usefulness of the water resources investigated. Although controlling and preventing most of the identified factors that could be contributing to the prevalence of medically important *Vibrio* species at the sampling points might be difficult, regular monitoring will go a long way to prevent possible *Vibrio*-related infection outbreaks.

## Data Availability Statement

The original contributions presented in the study are included in the article/Supplementary Material, further inquiries can be directed to the corresponding author.

## Author Contributions

OA and AIO initiated the research topic. AIO provided the materials for the study. OA structured the methods and carried out the statistical analysis and wrote the manuscript. OA and ACO carried out the experiment. ACO and AIO proofread and corrected the manuscript. All authors contributed to the article and approved the submitted version.

### Conflict of Interest

The authors declare that the research was conducted in the absence of any commercial or financial relationships that could be construed as a potential conflict of interest.
