# Supplementary material for: Molecular Detection and Distribution of Six Medically Important Vibrio spp. in Selected Freshwater and Brackish Water Resources in Eastern Cape Province, South Africa
Source: Front Microbiol. 2021 Jun 2;12:617703. doi: 10.3389/fmicb.2021.617703 (PMC8208477; doi:10.3389/fmicb.2021.617703)
Supplement: Supplementary file 1 [file Data_Sheet_1.docx]

TableSS1a: Yellow and green colony counts per sampling site (CFU/mL)

| Seasons | Colony color | Sampling sites | | | | | | | | | | | | | | | | |
| --- | --- | --- | --- | --- | --- | --- | --- | --- | --- | --- | --- | --- | --- | --- | --- | --- | --- | --- |
|  |  | PA1 | PA2 | PA3 | PA4 | PA5 | PA6 | PA7 | EL1 | EL2 | EL3 | EL4 | EL5 | EL6 | SR | SKR | ALD1 | ALD2 |
| Summer | Green | 2.5E+01 | 5.2E+02 | 9.5E+02 | 4.5E+02 | 2.3E+02 | 8.9E+02 | 1.5E+04 | 1.0E+04 | 8.8E+03 | 1.5E+04 | 1.8E+04 | 5.1E+04 | 1.2E+05 | 7.7E+02 | 5.3E+03 | 1.6E+02 | 3.2E+02 |
|  | Yellow | 4.5E+01 | 9.4E+02 | 1.7E+03 | 8.1E+02 | 4.2E+02 | 1.6E+03 | 3.1E+03 | 1.8E+04 | 1.6E+04 | 2.7E+04 | 3.2E+04 | 1.0E+04 | 2.4E+04 | 1.6E+02 | 1.1E+03 | 2.9E+02 | 5.9E+02 |
|  | Green | 8.4E+01 | 5.6E+01 | 1.2E+02 | 4.5E+01 | 8.0E+01 | 5.5E+01 | 1.4E+02 | 6.8E+01 | 1.9E+02 | 7.9E+01 | 5.4E+02 | 6.3E+02 | 1.5E+04 | ND | ND | ND | ND |
|  | Yellow | 1.5E+02 | 1.0E+02 | 2.0E+02 | 8.1E+01 | 1.5E+02 | 9.9E+01 | 2.9E+01 | 1.2E+02 | 3.4E+02 | 1.4E+02 | 9.6E+02 | 1.3E+02 | 3.0E+03 | ND | ND | ND | ND |
|  | Green | 2.5E+02 | 1.3E+02 | 5.6E+02 | 5.2E+02 | 2.6E+02 | 7.1E+03 | 2.7E+03 | 1.0E+02 | 1.1E+02 | 5.7E+02 | 1.3E+02 | 4.4E+02 | 2.8E+03 | 2.9E+03 | 2.3E+03 | 1.2E+01 | 5.2E+01 |
|  | Yellow | 4.5E+02 | 2.3E+02 | 1.0E+03 | 9.4E+02 | 4.7E+02 | 1.3E+04 | 5.6E+02 | 1.8E+02 | 2.0E+02 | 1.0E+03 | 2.3E+02 | 9.0E+01 | 5.9E+02 | 6.0E+02 | 4.7E+02 | 2.1E+01 | 9.4E+01 |
|  | Green | 1.8E+01 | 1.4E+02 | 8.2E+02 | 1.8E+03 | 3.4E+03 | 2.5E+03 | 2.0E+02 | 1.1E+01 | 1.5E+01 | 1.1E+03 | 5.2E+01 | 6.2E+02 | 7.6E+02 | 5.7E+03 | 1.3E+03 | 1.8E+01 | 1.7E+01 |
|  | Yellow | 3.2E+01 | 2.6E+02 | 1.5E+03 | 3.2E+03 | 6.1E+03 | 4.4E+03 | 3.8E+01 | 1.9E+01 | 2.8E+01 | 2.0E+03 | 9.4E+01 | 1.3E+02 | 1.5E+02 | 1.2E+03 | 2.7E+02 | 3.2E+01 | 3.0E+01 |
|  | Green | 4.0E+01 | 9.2E+01 | 4.9E+01 | 2.7E+02 | 6.7E+01 | 5.7E+01 | 1.2E+02 | ND | ND | ND | ND | ND | ND | 5.8E+01 | 5.9E+02 | 1.2E+02 | 1.5E+01 |
|  | Yellow | 7.3E+01 | 1.7E+02 | 8.8E+01 | 4.9E+02 | 1.2E+02 | 1.0E+02 | 2.5E+01 | ND | ND | ND | ND | ND | ND | 1.2E+01 | 1.2E+02 | 2.2E+02 | 2.7E+01 |
| Autumn | Green | 2.4E+01 | 2.0E+02 | 1.1E+02 | 1.3E+02 | 2.4E+01 | 1.8E+02 | 1.6E+02 | 7.6E+00 | 1.5E+01 | 2.1E+01 | 1.2E+01 | 2.2E+02 | 2.4E+03 | 3.3E+01 | 4.2E+01 | 7.4E+00 | 7.1E-01 |
|  | Yellow | 4.3E+01 | 3.7E+02 | 2.1E+02 | 2.4E+02 | 4.3E+01 | 3.2E+02 | 3.3E+01 | 1.4E+01 | 2.6E+01 | 3.8E+01 | 2.1E+01 | 4.6E+01 | 5.0E+02 | 6.8E+00 | 8.2E+00 | 1.3E+01 | 1.3E+00 |
|  | Green | 9.5E+00 | 3.4E+02 | 8.3E+01 | 2.4E+01 | 3.3E+01 | 8.8E+01 | 4.8E+01 | 4.5E+00 | 8.0E+00 | 4.7E+01 | 1.3E+01 | 7.1E+02 | 6.5E+02 | 2.9E+02 | 1.9E+02 | 4.8E+01 | 5.1E+00 |
|  | Yellow | 1.7E+01 | 6.2E+02 | 1.5E+02 | 4.3E+01 | 6.0E+01 | 1.6E+02 | 9.9E+00 | 8.1E+00 | 1.4E+01 | 8.4E+01 | 2.4E+01 | 1.5E+02 | 1.3E+02 | 6.0E+01 | 3.8E+01 | 8.6E+01 | 9.2E+00 |
| Winter | Green | 2.5E+01 | 5.9E+02 | 3.1E+02 | 3.7E+01 | 1.8E+01 | 1.0E+02 | 5.3E+01 | 6.7E+01 | 5.8E+01 | 5.0E+02 | 6.4E+02 | 2.2E+02 | 4.0E+02 | 3.1E+01 | 2.5E+02 | 5.4E+00 | 1.6E+01 |
|  | Yellow | 4.5E+01 | 1.1E+03 | 5.6E+02 | 6.7E+01 | 3.2E+01 | 1.8E+02 | 1.1E+01 | 1.2E+02 | 1.1E+02 | 9.0E+02 | 1.2E+03 | 4.5E+01 | 8.1E+01 | 6.1E+00 | 5.1E+01 | 9.6E+00 | 2.9E+01 |
|  | Green | 4.7E+01 | 2.9E+02 | 5.0E+01 | 3.1E+01 | 2.0E+01 | 3.6E+01 | 9.7E+01 | 2.7E+01 | 6.1E+00 | 1.8E+03 | 2.7E+01 | 1.7E+03 | 1.1E+02 | 1.3E+02 | 2.1E+01 | 9.6E-01 | 2.1E+00 |
|  | Yellow | 8.6E+01 | 5.3E+02 | 9.0E+01 | 5.5E+01 | 3.7E+01 | 6.4E+01 | 2.0E+01 | 4.8E+01 | 1.1E+01 | 3.1E+03 | 4.9E+01 | 3.5E+02 | 2.1E+01 | 2.6E+01 | 4.3E+00 | 1.7E+00 | 3.9E+00 |
|  | Green | 6.3E+01 | 1.4E+03 | 3.1E+02 | 4.3E+01 | 1.4E+02 | 5.5E+01 | 1.1E+02 | 1.6E+01 | 1.4E+01 | 7.7E+01 | 2.4E+01 | 1.5E+02 | 3.3E+01 | 3.0E+02 | 2.0E+02 | 4.2E+00 | 1.3E+00 |
|  | Yellow | 1.1E+02 | 2.5E+03 | 5.6E+02 | 7.7E+01 | 2.6E+02 | 9.9E+01 | 2.2E+01 | 2.9E+01 | 2.4E+01 | 1.4E+02 | 4.3E+01 | 3.0E+01 | 6.8E+00 | 6.3E+01 | 4.2E+01 | 7.5E+00 | 2.4E+00 |
| Spring | Green | 1.5E+01 | 2.7E+02 | 2.0E+02 | 1.5E+02 | 6.1E+01 | 1.4E+01 | 9.4E+01 | 2.6E+01 | 9.5E+00 | 5.3E+01 | 6.9E+01 | 2.2E+02 | 1.3E+02 | 3.3E+01 | 9.1E+01 | <1 | <1 |
|  | Yellow | 2.8E+01 | 4.9E+02 | 3.5E+02 | 2.7E+02 | 1.1E+02 | 2.6E+01 | 1.9E+01 | 4.8E+01 | 1.7E+01 | 9.4E+01 | 1.2E+02 | 4.6E+01 | 2.6E+01 | 6.7E+00 | 1.9E+01 | 1.3E+00 | 1.1E+00 |
|  | Green | 7.3E+00 | 1.4E+02 | 1.9E+02 | 2.2E+02 | 1.1E+02 | 8.0E+01 | 5.3E+01 | 6.1E+01 | 1.4E+01 | 2.9E+01 | 1.3E+01 | 1.0E+03 | 1.1E+02 | 2.2E+02 | 2.5E+02 | 8.3E-01 | 1.7E+00 |
|  | Yellow | 1.3E+01 | 2.5E+02 | 3.4E+02 | 4.1E+02 | 2.0E+02 | 1.4E+02 | 1.1E+01 | 1.1E+02 | 2.6E+01 | 5.1E+01 | 2.4E+01 | 2.1E+02 | 2.2E+01 | 4.5E+01 | 5.1E+01 | 1.5E+00 | 3.0E+00 |

Key: ND = Not determined (Samples were not collected because of student unrest)

Table SS1b: Density of the targeted *Vibrio* species in water samples

| Sites | LogMPN/ml | SD | Months | Sp |
| --- | --- | --- | --- | --- |
| PA1 | 0.866 | 0.3105 | $Jan | Vc |
| PA1 | 0.962 | 0.3179 | $March | Vc |
| PA1 | 0.968 | 0.2508 | $Dec | Vc |
| PA1 | 0.977 | 0.31187 | $Nov | Vc |
| PA1 | 1.166 | 0.2655 | $Feb | Vc |
| PA1 | 0.785 | 0.3071 | $Dec | Vf |
| PA1 | 0.977 | 0.31187 | $Nov | Vf |
| PA1 | 0.977 | 0.31187 | $March | Vf |
| PA1 | 1.056 | 0.2551 | $Jan | Vf |
| PA1 | 1.166 | 0.2655 | $Feb | Vf |
| PA2 | 0.977 | 0.31187 | $Nov | Va |
| PA2 | 0.866 | 0.3105 | $Feb | Vc |
| PA2 | 1.166 | 0.2655 | $Jan | Vc |
| PA2 | 1.29 | 0.1997 | $Dec | Vc |
| PA2 | 1.627 | 0.325 | $Nov | Vc |
| PA2 | 2.323 | 0.2357 | $March | Vc |
| PA2 | 1.056 | 0.2551 | $Feb | Vm |
| PA2 | 1.166 | 0.2655 | $Nov | Vm |
| PA2 | 1.187 | 0.2222 | $Dec | Vm |
| PA2 | 1.362 | 0.3099 | $Jan | Vm |
| PA2 | 2.166 | 0.2655 | $March | Vm |
| PA3 | 0.866 | 0.3105 | $Nov | Va |
| PA3 | 0.785 | 0.3071 | $Feb | Vc |
| PA3 | 0.962 | 0.3179 | $Jan | Vc |
| PA3 | 1.166 | 0.2655 | $Nov | Vc |
| PA3 | 1.427 | 0.2136 | $Dec | Vc |
| PA3 | 1.455 | 0.2161 | $March | Vc |
| PA3 | 0.785 | 0.3071 | $Feb | Vm |
| PA3 | 1.166 | 0.2655 | $Nov | Vm |
| PA3 | 1.166 | 0.2655 | $March | Vm |
| PA3 | 1.179 | 0.2219 | $Dec | Vm |
| PA3 | 1.627 | 0.2736 | $Jan | Vm |
| PA4 | 1.44 | 0.2148 | $Nov | Va |
| PA4 | 1.166 | 0.2655 | $Feb | Vc |
| PA4 | 1.289 | 0.1996 | $Dec | Vc |
| PA4 | 1.625 | 0.185 | $Jan | Vc |
| PA4 | 1.627 | 0.325 | $Nov | Vc |
| PA4 | 2.362 | 0.3099 | $March | Vc |
| PA4 | 1.166 | 0.2655 | $Feb | Vm |
| PA4 | 1.186 | 0.2221 | $Dec | Vm |
| PA4 | 1.32 | 0.2357 | $Nov | Vm |
| PA4 | 1.396 | 0.2114 | $Jan | Vm |
| PA4 | 1.963 | 0.3157 | $March | Vm |
| PA5 | 0.552 | 0.4365 | $April | Vc |
| PA5 | 1.048 | 0.2549 | $May | Vc |
| PA5 | 1.362 | 0.3099 | $Sept | Vc |
| PA5 | 1.539 | 0.1985 | $Dec | Vc |
| PA5 | 1.646 | 0.2655 | $Nov | Vc |
| PA5 | 1.963 | 0.3157 | $Jan | Vc |
| PA5 | 2.362 | 0.3099 | $Oct | Vc |
| PA5 | 2.963 | 0.3158 | $Feb | Vc |
| PA5 | 2.963 | 0.3158 | $March | Vc |
| PA5 | 1.166 | 0.2655 | $Dec | Vm |
| PA5 | 1.627 | 0.2736 | $Jan | Vm |
| PA5 | 1.627 | 0.325 | $March | Vm |
| PA5 | 1.869 | 0.3028 | $Nov | Vm |
| PA5 | 2.362 | 0.3099 | $Feb | Vm |
| PA6 | 1.556 | 0.1999 | $Dec | Vc |
| PA6 | 1.627 | 0.325 | $Sept | Vc |
| PA6 | 1.646 | 0.2655 | $Nov | Vc |
| PA6 | 2.31 | 0.2346 | $Jan | Vc |
| PA6 | 2.362 | 0.3099 | $Feb | Vc |
| PA6 | 2.628 | 0.3251 | $Oct | Vc |
| PA6 | 2.72 | 0.174 | $March | Vc |
| PA7 | 1.155 | 0.2647 | $March | Va |
| PA7 | 1.179 | 0.2219 | $Jan | Va |
| PA7 | 1.362 | 0.3099 | $Feb | Va |
| PA7 | 1.862 | 0.302 | $Dec | Va |
| PA7 | 0.589 | 0.859 | $Nov | Va |
| PA7 | 0.553 | 0.4365 | $Nov | Vc |
| PA7 | 0.865 | 0.3105 | $Jan | Vc |
| PA7 | 0.962 | 0.3179 | $March | Vc |
| PA7 | 0.977 | 0.31187 | $Dec | Vc |
| PA7 | 1.362 | 0.3099 | $Feb | Vc |
| PA7 | 0.962 | 0.3179 | $Jan | Vm |
| PA7 | 0.962 | 0.3179 | $Feb | Vm |
| PA7 | 1.056 | 0.2551 | $March | Vm |
| PA7 | 1.426 | 0.2135 | $Dec | Vm |
| PA7 | 0.589 | 0.859 | $Nov | Vm |
| PA7 | 0.785 | 0.3071 | $Dec | Vp |
| PA7 | 0.962 | 0.3179 | $Feb | Vp |
| PA7 | 1.155 | 0.2647 | $March | Vp |
| PA7 | 1.179 | 0.2219 | $Jan | Vp |
| PA7 | 0.589 | 0.859 | $Nov | Vp |
| EL1 | 0.977 | 0.31187 | $Dec | Vm |
| EL1 | 1.056 | 0.2551 | $Jan | Vm |
| EL1 | 1.056 | 0.2551 | $Feb | Vm |
| EL1 | 1.525 | 0.1975 | $Nov | Vm |
| EL2 | 1.362 | 0.3099 | $Nov | Va |
| EL2 | 0.552 | 0.4365 | $April | Vm |
| EL2 | 0.552 | 0.4365 | $May | Vm |
| EL2 | 1.166 | 0.2655 | $Dec | Vm |
| EL2 | 1.362 | 0.3099 | $Jan | Vm |
| EL2 | 1.627 | 0.325 | $Nov | Vm |
| EL2 | 1.627 | 0.325 | $Feb | Vm |
| EL3 | 1.04 | 0.2547 | $Dec | Vc |
| EL3 | 1.166 | 0.2655 | $Feb | Vc |
| EL3 | 1.362 | 0.3099 | $Jan | Vc |
| EL3 | 1.794 | 0.2906 | $Nov | Vc |
| EL3 | 1.166 | 0.2655 | $Feb | Vf |
| EL3 | 1.272 | 0.1992 | $Dec | Vf |
| EL3 | 1.627 | 0.325 | $Nov | Vf |
| EL3 | 2.97 | 0.3099 | $Jan | Vf |
| EL3 | 0.866 | 0.3105 | $Nov | Vv |
| EL3 | 0.866 | 0.3105 | $Dec | Vv |
| EL3 | 1.056 | 0.2551 | $Feb | Vv |
| EL3 | 1.166 | 0.2655 | $Jan | Vv |
| EL4 | 0.962 | 0.3179 | $Nov | Vc |
| EL4 | 1.048 | 0.2549 | $Jan | Vc |
| EL4 | 1.31 | 0.2346 | $Dec | Vc |
| EL4 | 1.643 | 0.1862 | $Feb | Vc |
| EL5 | 0.962 | 0.3179 | $April | Va |
| EL5 | 1.04 | 0.2547 | $Nov | Va |
| EL5 | 1.04 | 0.2547 | $Dec | Va |
| EL5 | 1.195 | 0.2224 | $Jan | Va |
| EL5 | 1.362 | 0.3099 | $Feb | Va |
| EL5 | 1.627 | 0.325 | $May | Va |
| EL5 | 0.552 | 0.4365 | $Jan | Vc |
| EL5 | 0.552 | 0.4365 | $Feb | Vc |
| EL5 | 0.962 | 0.3179 | $April | Vc |
| EL5 | 1.166 | 0.2655 | $July | Vc |
| EL5 | 1.166 | 0.2655 | $Aug | Vc |
| EL5 | 1.17 | 0.2217 | $Nov | Vc |
| EL5 | 1.171 | 0.2217 | $Dec | Vc |
| EL5 | 1.627 | 0.325 | $May | Vc |
| EL5 | 1.627 | 0.325 | $June | Vc |
| EL5 | 1.166 | 0.2655 | $July | Vf |
| EL5 | 1.166 | 0.2655 | $Aug | Vf |
| EL5 | 1.627 | 0.325 | $June | Vf |
| EL5 | 0.962 | 0.3179 | $Aug | Vm |
| EL5 | 1.627 | 0.325 | $June | Vm |
| EL5 | 1.627 | 0.325 | $July | Vm |
| EL5 | 0.962 | 0.3179 | $Feb | Vp |
| EL5 | 1.04 | 0.2547 | $Nov | Vp |
| EL5 | 1.17 | 0.2217 | $Dec | Vp |
| EL5 | 1.195 | 0.2224 | $Jan | Vp |
| EL6 | 0.552 | 0.4365 | $May | Va |
| EL6 | 0.859 | 0.3103 | $Jan | Va |
| EL6 | 1.166 | 0.2655 | $Feb | Va |
| EL6 | 1.31 | 0.2346 | $April | Va |
| EL6 | 1.44 | 0.2148 | $Nov | Va |
| EL6 | 1.44 | 0.2148 | $Dec | Va |
| EL6 | 0.778 | 0.307 | $Dec | Vm |
| EL6 | 0.859 | 0.3103 | $Jan | Vm |
| EL6 | 1.166 | 0.2655 | $Feb | Vm |
| EL6 | 1.31 | 0.2346 | $Nov | Vm |
| EL6 | 0.477 | 0.4342 | $Dec | Vp |
| EL6 | 0.552 | 0.4365 | $May | Vp |
| EL6 | 0.859 | 0.3103 | $Jan | Vp |
| EL6 | 0.866 | 0.3105 | $April | Vp |
| EL6 | 0.962 | 0.3179 | $July | Vp |
| EL6 | 1.155 | 0.2647 | $Nov | Vp |
| EL6 | 1.166 | 0.2655 | $Feb | Vp |
| EL6 | 1.362 | 0.3099 | $Aug | Vp |
| EL6 | 1.627 | 0.325 | $June | Vp |
| SR | 0.484 | 0.4342 | $Nov | Va |
| SR | 0.785 | 0.3071 | $March | Va |
| SR | 1.28 | 0.1994 | $Jan | Va |
| SR | 2.87 | 0.3029 | $Feb | Va |
| SR | 0.484 | 0.4342 | $Nov | Vc |
| SR | 1.047 | 0.2549 | $Jan | Vc |
| SR | 1.323 | 0.2357 | $March | Vc |
| SR | 2.166 | 0.2655 | $Feb | Vc |
| SR | 0.484 | 0.4342 | $Nov | Vm |
| SR | 0.866 | 0.3105 | $Jan | Vm |
| SR | 1.048 | 0.2549 | $March | Vm |
| SR | 1.056 | 0.2551 | $Feb | Vm |
| SR | 0.785 | 0.3071 | $Nov | Vp |
| SR | 0.866 | 0.3105 | $Jan | Vp |
| SR | 0.962 | 0.2507 | $March | Vp |
| SR | 1.963 | 0.3157 | $Feb | Vp |
| SR | 0.484 | 0.4342 | $Nov | Vv |
| SR | 0.866 | 0.3105 | $Jan | Vv |
| SR | 1.166 | 0.2655 | $March | Vv |
| SR | 1.362 | 0.3099 | $Feb | Vv |
| SKR | 0.866 | 0.3105 | $Nov | Va |
| SKR | 0.977 | 0.31187 | $March | Va |
| SKR | 1.166 | 0.2655 | $April | Va |
| SKR | 1.362 | 0.3099 | $May | Va |
| SKR | 1.627 | 0.325 | $Feb | Va |
| SKR | 2.166 | 0.2655 | $Jan | Va |
| SKR | 0.962 | 0.3179 | $April | Vc |
| SKR | 0.977 | 0.31187 | $Nov | Vc |
| SKR | 1.166 | 0.2655 | $March | Vc |
| SKR | 1.362 | 0.3099 | $May | Vc |
| SKR | 1.627 | 0.325 | $Feb | Vc |
| SKR | 2.166 | 0.2655 | $Jan | Vc |
| SKR | 1.362 | 0.3099 | $July | Vf |
| SKR | 1.627 | 0.325 | $Aug | Vf |
| SKR | 2.166 | 0.2655 | $June | Vf |
| SKR | 0.866 | 0.3105 | $Feb | Vp |
| SKR | 0.962 | 0.3179 | $Nov | Vp |
| SKR | 1.166 | 0.2655 | $March | Vp |
| SKR | 1.166 | 0.2655 | $April | Vp |
| SKR | 1.362 | 0.3099 | $May | Vp |
| SKR | 1.627 | 0.325 | $Jan | Vp |
| ALD1 | 0.484 | 0.4342 | $Jan | Vf |
| ALD1 | 0.866 | 0.3105 | $Nov | Vf |
| ALD1 | 1.166 | 0.2655 | $Feb | Vf |
| ALD1 | 1.166 | 0.2655 | $March | Vf |
| ALD2 | 0.792 | 0.3071 | $March | Vc |
| ALD2 | 1.627 | 0.325 | $Jan | Vc |
| ALD2 | 1.627 | 0.325 | $Feb | Vc |
| ALD2 | 1.963 | 0.3157 | $Nov | Vc |
| ALD2 | 0.484 | 0.4342 | $July | Vp |
| ALD2 | 1.056 | 0.2551 | $June | Vp |
| ALD2 | 1.166 | 0.2655 | $Aug | Vp |

Key: Vc = *V. cholerae,* Vm = *V. mimicus,* Vf = *V. fluvialis,* Vv = *V. vulnificus,* Va = *V. alginolyticus and* Vp = *V. parahaemolyticus*

Note: Only sites with detectable density (> 0.477 LogMPN/mL) of the targeted species are presented.

A

B

Figure SS1A&B: Influence of temperature and salinity on the absolute density of *Vibrio* spp. in water samples.

Key: A = Freshwater samples, B= Brackish water samples

Key: Va= *V. alginolyticus,* VC = *V. cholerae*, Vf = *V. fluvialis*, Vm = *V. mimicus*, Vp = *V. parahaemolyticus*, Vv = *V. vulnificus*, ATS = all targeted vibrio species.

Table SS1C: Correlation analysis between the absolute densities of the targeted *Vibrio* spp. from water samples, temperature and salinity

| Freshwater | | | | Brackish water | | | |
| --- | --- | --- | --- | --- | --- | --- | --- |
| Organism type | Parameters | R | P values | Organism type | Parameter | R | P values |
| FITV | Sal | **0.215** | **0.001** | FITV | Sal | -0.030 | 0.582 |
|  | Temp | **0.114** | **<0.001** |  | Temp | **0.200** | **<0.0001** |
| FIVc | Sal | **0.382** | **<0.0001** | FIVc | Sal | -0.031 | 0.818 |
|  | Temp | **0.501** | **<0.0001** |  | Temp | **0.490** | **<0.0001** |
| FIVm | Sal | **0.174** | **0.041** | FIVm | Sal | 0.055 | 0.686 |
|  | Temp | **0.260** | **0.002** |  | Temp | 0.032 | 0.816 |
| FIVf | Sal | -0.168 | 0.049 | FIVf | Sal | -0.245 | 0.068 |
|  | Temp | **0.176** | **0.039** |  | Temp | 0.212 | 0.116 |
| FIVa | Sal | -0.045 | 0.600 | FIVa | Sal | -0.153 | 0.259 |
|  | Temp | -0.12 | 0.888 |  | Temp | **0.500** | **<0.0001** |
| FIVv | Sal | 0.001 | 0.987 | FIVv | Sal | -0.321 | 0.016 |
|  | Temp | 0.057 | 0.505 |  | Temp | **0.382** | **0.004** |
| FIVp | Sal | -0.088 | 0.305 | FIVp | Sal | -0.131 | 0.335 |
|  | Temp | _-0.137 | 0.108 |  | Temp | **0.571** | **<0.0001** |

Key: Bolded R and P values show a significant correlation, FIA=Frequency of isolation; TV = the targeted *Vibrio* spp.; VA= *V. alginolyticus,* VC=*V. cholerae*,VF= *V. fluvialis*,VM= *V. mimicus*,VP= *V. parahaemolyticus*,VV = *V. vulnificus,* %P = percentage prevalence

Table SS1d: Comparison of absolute density of targeted *Vibrio* spp. in samples from freshwater and brackish water samples

| Species | Sites comparison that showed significant difference |
| --- | --- |
| *V. cholerae* | PA1<PA5 (p=0.001), PA1<PA6(p=0.004),PA2<PA5 (p=0.008),PA2<PA6 (p=0.026), PA2>EL1(p=0.036),PA2>EL2(p=0.036),PA2>EL6(p=0.036),PA2>ALD1(p=0.036), PA3<PA5 (p=0.002), PA3<PA6 (p=0.008), PA4<PA5 (p=0.015), PA4<PA6(p=0.046), PA4>EL1(P=0.02), PA4>EL2(P=0.02), PA4>EL6(P=0.02), PA5>PA7(p=0.001), PA5>EL1(p<0.0001), PA5>EL2(p<0.0001), PA5>EL3(p=0.003), PA5>EL4(p=0.002), PA5>EL6(p<0.001), PA5>SKR(p=0.034), PA5>SR(p=0.005),PA5>ALD1(p<0.001), PA5>ALD2(p<0.005), PA6>PA7(p=0.003), PA6>EL1(p<0.0001), PA6>EL2(p<0.0001), PA6>EL3(p<0.01), PA6>EL4(p<0.007), PA6>EL6(p<0.0001), PA6>SR(p=0.017),PA6>ALD1(p<0.0001), PA6>ALD2(p<0.017), EL1<EL5(p=0.002), EL1<SKR(p=0.011), EL2<EL5(p=0.002), EL2<SKR(p=0.011), EL5>EL6(p=0.002), EL5>ALD1(p=0.002), EL6<SKR(p=0.011), SKR>ALD1(p=0.011), |
| *V. mimicus* | PA1<PA3(p=0.007), PA2<PA3(p=0.031), PA3>PA4(p=0.037), PA3>PA5(p=0.017), PA3>PA4(p=0.037) PA3>PA6(p=0.003), PA3>EL1(p=0.02), PA3>EL2(p=0.027), PA3>EL3(p=0.004), PA3>EL4(p=0.004), PA3>EL5(p=0.016), PA3>EL6(p=0.016), PA3>SKR(p=0.004), PA3>ALD1(p=0.004), PA3>ALD2(p=0.004) |
| *V. fluvialis* | PA1>PA2(p=0.009), PA1>PA3(p=0.009),PA1>PA3(p=0.009) PA1>PA4(p=0.009), PA1>PA5(p=0.009), PA1>PA6(p=0.009), PA1>PA7(p=0.009), PA1>EL1(p=0.011), PA1>EL2(p=0.011), PA1>EL4(p=0.011), PA1>EL6(p=0.011), PA1>SR(p=0.011), PA1>EL2(p=0.011), PA2<EL3(p<0.001), PA2<EL5(p=0.026), PA2<SKR(p=0.004), PA2<ALD1(p=0.038), PA3<EL3(p<0.001), PA3<EL5(p=0.026), PA3<SKR(p=0.004), PA3<ALD1(p=0.038), PA4<EL3(p<0.001), PA4<EL5(p=0.026), PA4<SKR(p=0.004), PA4<ALD1(p=0.038), PA5<EL3(p<0.001), PA5<EL5(p=0.026), PA5<SKR(p=0.004), PA5<ALD1(p=0.038), PA6<EL3(p<0.001), PA6<EL5(p=0.026), PA6<SKR(p=0.004), PA6<ALD1(p=0.038), PA7<EL3(p<0.001), PA7<EL5(p=0.026), PA7<SKR(p=0.004), PA7<ALD1(p=0.038), EL1<EL3(p<0.001), EL1<EL5(p=0.029), EL1<SKR(p=0.005), EL1<ALD1(p=0.042),EL2<EL3(p<0.001), EL2<EL5(p=0.029), EL2<SKR(p=0.005), EL2<ALD1(p=0.042), EL3>EL4(p<0.001), EL3>EL6(p<0.001), EL3>SR(p<0.001), EL3>ALD2(p<0.001), EL4<EL5(p=0.029), EL4<SKR(p=0.005), EL4<ALD1(p=0.042), EL5>EL6(p=0.029), EL5>SR(p=0.029), EL5>ALD2(p=0.029), EL6<SKR(p=0.005), EL6<ALD1(p=0.042), SKR>SR(p=0.005), SKR>ALD2(p=0.005), SR>ALD1(p=0.042), ALD1>ALD2(0.042) |
| *V. vulnificus* | PA1<EL3(p<0.001), PA1<SR(p<0.001), PA2<EL3(p<0.001), PA2<SR(p<0.001),PA3<EL3(p<0.001), PA3<SR(p<0.001), PA4<EL3(p<0.001), PA4<SR(p<0.001), PA5<EL3(p<0.001), PA5<SR(p<0.001), PA6<EL3(p<0.001), PA1<SR(p<0.001), EL1<EL3(p<0.001), EL1<SR(p<0.001), EL2<EL3(p<0.001), EL2<SR(p<0.001), EL3>EL4(p<0.001), EL3>EL5(p<0.001), EL3>EL6(p<0.001), EL3>SKR(p<0.001), EL3>ALD1(p<0.001), EL3>ALD2(p<0.001), EL4<SR(<0.001), EL5<SR(<0.001), EL6<SR(<0.001), EL4<SKR(<0.001), SR>ALD1(<0.001), SR>ALD2(<0.001) |
| *V. alginolyticus* | PA7>PA1 (p=0.013), PA7>PA2 (p=0.013), PA7>PA3 (p=0.026), PA7>PA4 (p=0.064), PA7>PA5 (p=0.013), PA7>PA6 (p=0.013), PA7>EL1 (p=0.015), PA7>EL2 (p=0.070), PA7>EL3 (p=0.015), PA7>EL4 (p=0.015), PA7>ALD1 (p=0.015), PA7>ALD2 (p=0.015),EL5>PA1 (p=0.002), EL5>PA2 (p=0.006), EL5>PA3 (p=0.005), EL5>PA4 (p=0.014), EL5>PA5 (p=0.002), EL5>PA6 (p=0.002), EL5>EL1 (p=0.003), EL5>EL2 (p=0.016), EL5>EL3 (p=0.003), EL5>EL4 (p=0.003), EL5>ALD1 (p=0.003), EL5>ALD2 (p=0.003), EL6>PA1 (p=0.006), EL6>PA2 (p=0.015), EL6>PA3 (p=0.012), EL6>PA4 (p=0.033), EL6>PA5 (p=0.006), EL6>PA6 (p=0.006), EL6>EL1 (p=0.007), EL6>EL2 (p=0.037), EL6>EL3 (p=0.007), EL6>EL4 (p=0.007), EL6>ALD1 (p=0.007),EL6>ALD2 (p=0.007), SR>PA1 (p=0.013), SR>PA2 (p=0.031), SR>PA3 (p=0.026), SR>PA4 (p=0.063), SR>PA5 (p=0.013), SR>PA6 (p=0.013), SR>EL1 (p=0.015), SR>EL3 (p=0.015), SR>EL4 (p=0.015), SR>ALD1 (p=p=0.015),SR>ALD2 (p=p=0.015), SKR>PA1 (p<0.001), SKR>PA2 (p=0.001), SKR>PA3 (p=0.001), SKR>PA4 (p=0.002), SKR>PA5 (p<0.001), SKR>PA6 (p<0.001), SKR>EL1 (p<0.001), SKR>EL2 (p=0.002), SKR>EL3 (p<0.001), SKR>EL4 (p<0.001), SKR>ALD1 (p=p<0.001),SKR>ALD2 (p=p<0.001) |
| *V. parahaemolyticus* | PA1<PA7(p<0.001), PA1<EL6(p=0.001), PA1<SKR(p=0.001), PA1<SR(p=0.031),PA2<PA7(p<0.001), PA2<EL6(p=0.001), PA2<SKR(p=0.001), PA2<SR(p=0.031), PA3<PA7(p<0.001), PA3<EL6(p=0.001), PA3<SKR(p=0.001), PA3<SR(p=0.031), PA4<PA7(p<0.001), PA4<EL6(p=0.001), PA4<SKR(p=0.001), PA4<SR(p=0.031), PA5<PA7(p<0.001), PA5<EL6(p=0.001), PA5<SKR(p=0.001), PA5<SR(p=0.031), PA6<PA7(p<0.001), PA6<EL6(p=0.001), PA6<SKR(p=0.001), PA6<SR(p=0.031), PA7>EL1(p<0.001), PA7>EL2(p<0.001), PA7>EL3(p<0.001), PA7>EL4(p<0.001), PA7>EL5(p=0.03), PA7>ALD1(p<0.001), PA7>ALD2(p=0.006), EL1<EL6(p=0.001), EL1<SKR(p=0.011), EL1<SKR(p=0.035), EL2<EL6(p=0.001), EL2<SKR(p=0.011), EL2<SKR(p=0.035), EL3<EL6(p=0.001), EL3<SKR(p=0.011), EL3<SKR(p=0.035), EL4<EL6(p=0.001), EL4<SKR(p=0.011), EL4<SKR(p=0.035), EL6>ALD1(p=0.001), EL6>ALD2(p=0.025), SKR>ALD1(p=0.011), SR>ALD1(p=0.035) |

Table SS1e: Seasonal Comparison of mean absolute density of each of the targeted species per water type

| **Freshwater** | | | | **Brackish water** | | | |
| --- | --- | --- | --- | --- | --- | --- | --- |
| **Species** | **Season (I) VS Season (J)** | | **P values** | **Species** | **Season (I) VS Season (J)** | | **P values** |
| *V. cholerae* | **Summer** | Autumn | **<0.001** | *V. cholerae* | **Summer** | Autumn | **0.06** |
|  | **Summer** | Winter | **<0.001** |  | **Summer** | Winter | **0.004** |
|  | **Summer** | Spring | **<0.001** |  | **Summer** | Spring | **<0.001** |
|  | Autumn | Winter | 0.701 |  | Autumn | Winter | 0.485 |
|  | Autumn | Spring | 0.164 |  | Autumn | Spring | 0.059 |
|  | Winter | Spring | 0.057 |  | Winter | Spring | 0.163 |
| *V. mimicus* | **Summer** | Autumn | **0.022** | *V. mimicus* | **Summer** | Autumn | **0.02** |
|  | **Summer** | Winter | **0.007** |  | **Summer** | Winter | **0.041** |
|  | **Summer** | Spring | **0.17** |  | **Summer** | Spring | **0.02** |
|  | Autumn | Winter | 0.923 |  | Autumn | Winter | 0.606 |
|  | Autumn | Spring | 0.93 |  | Autumn | Spring | 1 |
|  | Winter | Spring | 1 |  | Winter | Spring | 0.606 |
| *V. fluvialis* | **Summer** | Autumn | **0.002** | *V. fluvialis* | Summer | Autumn | 1 |
|  | **Summer** | Winter | **<0.001** |  | **Summer** | Winter | **<0.001** |
|  | **Summer** | Spring | **0.002** |  | Summer | Spring | 1 |
|  | Autumn | Winter | 1 |  | **Autumn** | Winter | **0.001** |
|  | Autumn | Spring | 1 |  | Autumn | Spring | 1 |
|  | Winter | Spring | 1 |  | **Winter** | Spring | **0.001** |
| *V. vulnificus* | Summer | Autumn | 0.074 | *V. vulnificus* | Summer | Autumn | 0.069 |
|  | **Summer** | Winter | **0.42** |  | **Summer** | Winter | **0.04** |
|  | Summer | Spring | 0.074 |  | Summer | Spring | 0.069 |
|  | Autumn | Winter | 1 |  | Autumn | Winter | 1 |
|  | Autumn | Spring | 1 |  | Autumn | Spring | 1 |
|  | Winter | Spring | 1 |  | Winter | Spring | 1 |
| *V. alginolyticus* | **Summer** | Autumn | **<0.001** | *V. alginolyticus* | **Summer** | Autumn | **0.07** |
|  | **Summer** | Winter | **<0.001** |  | **Summer** | Winter | **<0.001** |
|  | **Summer** | Spring | **<0.001** |  | **Summer** | Spring | **<0.001** |
|  | Autumn | Winter | 1 |  | **Autumn** | Winter | **0.001** |
|  | Autumn | Spring | 1 |  | **Autumn** | Spring | **0.001** |
|  | Winter | Spring | 1 |  | Winter | Spring | 1 |
| *V. parahaemolyticus* | Summer | Autumn | 1 | *V. parahaemolyticus* | **Summer** | Autumn | **0.1** |
|  | Summer | Winter | **0.012** |  | **Summer** | Winter | **0.003** |
|  | Summer | Spring | 1 |  | **Summer** | Spring | **<0.001** |
|  | Autumn | Winter | **0.039** |  | Autumn | Winter | 0.917 |
|  | Autumn | Spring | 1 |  | Autumn | Spring | 0.351 |
|  | Winter | Spring | **0.039** |  | Winter | Spring | 0.361 |

Note: Significant comparison p values are in bold

Table SS1f: Distribution of isolated targeted species per sampling site

| **sites** | **PSVI** | **CVI** | **Vc** | **Vm** | **Vf** | **Va** | **Vv** | **Vp** | **OVSP** |
| --- | --- | --- | --- | --- | --- | --- | --- | --- | --- |
| PA1 | 49 | 37 | 2 |  | 1 | _ | _ | _ | 34 |
| PA2 | 61 | 44 | 4 | 2 | _ | 1 |  |  | 37 |
| PA3 | 65 | 50 | 3 | 2 | _ | 1 | _ | _ | 44 |
| PA4 | 68 | 55 | 9 | 4 | _ | 3 |  | _ | 39 |
| PA5 | 53 | 44 | 24 | 2 | _ | _ | _ | _ | 18 |
| PA6 | 68 | 60 | 33 | _ | _ | _ | _ | _ | 27 |
| PA7 | 50 | 35 | 3 | 2 | _ | 3 |  | 2 | 25 |
| EL1 | 43 | 35 |  | 2 | _ | _ | _ | _ | 33 |
| EL2 | 42 | 40 | _ | 3 | _ | 1 | _ | _ | 36 |
| EL3 | 58 | 39 | 1 | _ | 2 | _ | 1 | _ | 35 |
| EL4 | 53 | 40 | 3 |  | _ | _ | _ | _ | 37 |
| EL5 | 90 | 76 | 8 | 1 | 1 | 8 | _ | 1 | 57 |
| EL6 | 73 | 64 | _ | 3 |  | 17 | 1 | 4 | 39 |
| SR | 48 | 42 | 3 | 1 | 0 | 17 | 2 | 4 | 15 |
| SKR | 81 | 73 | 14 | 1 | 2 | 11 | 0 | 10 | 35 |
| ALD1 | 38 | 27 | 0 | _ | 1 | _ | _ | _ | 26 |
| ALD2 | 30 | 25 | 3 | _ | _ | _ | _ | 1 | 21 |

Key _ = not isolated, VA= *V. alginolyticus,* VC=*V. cholerae*,VF= *V. fluvialis*,VM= *V. mimicus*,VP= *V. parahaemolyticus*,VV = *V. vulnificus,* PSVI = presumptive vibrio species isolated, CVI = PCR-confirmed vibrio isolate, OVSP = other *Vibrio* species
